# Future global distribution and climatic suitability of *Anopheles stephensi*

**DOI:** 10.1038/s41598-025-07653-8

**Published:** 2025-07-01

**Authors:** Andre Luis Acosta, Marcia C. Castro, Gabriel Z. Laporta, Jan E. Conn, Maria Anice M. Sallum

**Affiliations:** 1 Epidemiology Department, Public Health School, Universidade de São Paulo (USP), São Paulo, 01246-904 SP Brazil; 2 Planetary Health Brazil, Institute of Advanced Studies, Universidade de São Paulo (USP), São Paulo, 05508-050 SP Brazil; 3 Department of Global Health and Population, Harvard T.H. Chan School of Public Health, Boston, MA 02115 USA; 4 Graduate Program in Health Sciences, FMABC Medical School University Center, Santo Andre, SP 09060-870 Brazil; 5 Wadsworth Center, New York State Department of Health, Albany, NY 12237 USA; 6 Department of Biomedical Sciences, College of Integrated Health Sciences, State University of New York-Albany, Albany, NY USA

**Keywords:** *Anopheles stephensi*, Urban malaria, Climate change, Habitat suitability models, Invasive species, Vector-borne diseases, Ecology, Climate sciences, Ecology, Planetary science, Diseases, Medical research

## Abstract

*Anopheles stephensi*, an urban malaria vector, is expanding into new regions and poses a growing global health threat. Using a robust multi-model framework with eight algorithms and high-resolution climate data, this study assessed and mapped current and future global climate suitability for the species, incorporating demographic data to estimate populations at risk in different scenarios. Currently, *An. stephensi* can thrive in 13% of the Earth’s surface, affecting nearly 40% of the global population. Projections indicate that suitable areas could exceed 30% by 2100, exposing up to 56% of the world’s population, including in previously malaria-free regions such as North America, Europe, and Oceania. Spread may occur via maritime traffic, wind dispersal, and natural range expansion. These findings highlight the urgent need for global monitoring and control strategies, emphasizing the importance of integrating climate change projections into urban malaria prevention efforts to protect vulnerable populations and support global malaria elimination goals.

**Supplementary Information:**

The online version contains supplementary material available at 10.1038/s41598-025-07653-8.

## Introduction

*Anopheles* (*Cellia*) *stephensi* is a mosquito that is found in sylvatic, rural, and urban areas. It is responsible for transmitting malaria throughout Southeast Asia, the Middle East, and the Arabian Peninsula^1,2^, and exhibits three distinct biological forms: type, intermediate, and mysorensis. These forms can be distinguished by external egg morphology^3^, mitochondrial DNA sequence data^4–8^, and nuclear genes^9–11^. The type and intermediate forms are capable of transmitting *Plasmodium falciparum* and *P*. *vivax*^12^. These forms are anthropophilic, preferring to feed and rest inside human dwellings^13^, but may also feed outdoors during the hot season when people sleep outside at night^14^. These behaviors increase the likelihood of vector-human contact and, consequently, the risk of human malaria transmission. Both the type and intermediate biological forms of *An*. *stephensi* can spread rapidly in human-dominated habitats in urban and peri-urban environments^15^. Female mosquitoes lay eggs and develop immature stages in various artificial containers, such as abandoned tires, water tanks, cisterns, wells, gutters, jars, and other utensils used for water storage near human dwellings^16^. Additionally, the immature stages of *An*. *stephensi* can survive in polluted water^17^ and brackish water habitats, as observed in India^18^ and Sri Lanka^19^. These biological and ecological characteristics collectively enhance the species’ capacity to invade, spread, and establish in new urban environments.

The geographic distribution of *An*. *stephensi* spans regions from southeastern China to India, Iraq, Iran, the Arabian Peninsula, and Egypt^19^. Recently, its range has expanded in African continent, particularly in the Horn of Africa. The invasion of *An*. *stephensi* was first identified in an area 20 km from the port of Djibouti in 2012^4^, followed by eastern Ethiopia and Sudan in 2016^6,20–22^, Somalia in 2019^23^, Somaliland in 2020^7^, and southern Ethiopia in 2022^24^. On the Atlantic coast of Africa, *An*. *stephensi* invaded Nigeria in 2020 and Ghana in 2021^25–27^. In Sri Lanka, *An*. *stephensi* was reported in 2017^28^, and in Yemen, the species was found in water containers in camps for internally displaced persons in 2021^29^.

Until recently, malaria in Africa was primarily a rural public health problem^26,27,30,31^. However, the invasion and spread of *An*. *stephensi* has boosted urban malaria transmission in countries where the species has become established^26,31,32^. The spread of *An*. *stephensi* poses a significant threat to malaria control and elimination efforts^15,21,33^, as 40% of the sub-Saharan population lives in densely populated urban areas. The recent spread of *An*. *stephensi* is a stark reminder of the deficiencies in current vector surveillance and control efforts^32^.

The establishment and spread of *An*. *stephensi* in Africa are likely influenced by a complex interplay of environmental and anthropogenic factors. Precarious construction practices amidst rapid and unplanned urban expansion create ideal larval habitats for this malaria vector, even during the dry season^34^. Although *An*. *stephensi* is primarily detected in urban areas, it can also proliferate in rural areas due to its ability to utilize various natural and artificial water reservoirs during larval stages^35^. This ecological flexibility allows the mosquito to move seamlessly between rural and urban settings, complicating control efforts. In addition, the effects of climate change may reduce water availability in rural areas, forcing human populations to migrate to the outskirts of cities^36^, often settling in impoverished squatter areas. Furthermore, immature stages can reach new locations via maritime transport (aboard ships), as well as adult mosquitoes that hatch during travel^31,37^. Modeling of maritime freight traffic in the Horn of Africa estimated that Djibouti and Sudan were the country’s most vulnerable to *An*. *stephensi* establishment^25^. Additionally, spread across Africa may be associated with wind-assisted long-distance migration^31^. This was documented in the Sahelian zone of Mali, where 235 anopheline mosquitoes were collected during 617 nocturnal aerial collections at 40–290 m above ground level^37^. Studies have shown that climatic factors critically shape both the expansion potential of *Anopheles stephensi* and the efficiency of malaria transmission. Transmission efficiency of *Plasmodium falciparum* and *P. vivax* via *Anopheles* mosquitoes is constrained to a thermal range of approximately 18–32 °C, with optimal transmission around 25 °C^38^. Below 18 °C, vector survival declines sharply, making sporogony completion unlikely^39^. Additionally, *An. gambiae* s.s. *-* a physiological proxy for *An. stephensi -* exhibits optimal aquatic development between 22 and 32 °C, with no emergence below 18 °C or above 34 °C^40^. High relative humidity (> 60%) is also critical to adult mosquito longevity and transmission success^39,41^. These thresholds are further supported by regional studies, such as Ayanlade et al. (2013)^42^, who demonstrated that intra-annual climate variability *-* particularly in temperature, humidity, and rainfall *-* has a decisive influence on malaria transmission dynamics in Nigeria.

Changes in climate affect the life cycles of mosquito vectors and malaria parasites, facilitating colonization and spread, and intensifying malaria transmission^43^. Projected thermal limits for future malaria occurrence suggest that the spread of *An*. *stephensi* into urban areas could significantly expand the landscape of malaria transmission due to thermal suitability for transmission of *P*. *falciparum* and *P*. *vivax*, potentially extending as far as the Arctic^44^. Understanding the rapid spread of *An*. *stephensi* in Africa is critical to mitigating the risk of its expansion into other regions^31^. A recent landscape genomic study by Samake et al. (2023)^35^ revealed that genetic drift, followed by founder events, are the primary factors shaping the genetic variation of *An. stephensi* populations in the Horn of Africa. Once established in urban ecosystems, natural selection processes will further influence genetic variation in mosquito populations. This process, potentially accelerated by climate change, could permanently urbanize vector populations and consequently intensify malaria transmission in cities, leading to a catastrophic scenario.

Here we aim to assess and map the global climate suitability for *An*. *stephensi* from an empirical baseline climate scenario (1970–2000) to projected future scenarios extending to 2100. Our findings underscore the escalating malaria threat posed by *An*. *stephensi* to global public health and malaria elimination goals^21,26,27^, highlighting the urgency of increasing entomological surveillance in areas at risk of species colonization.

## Results

Climate suitability projections were generated using ensemble forecast models (EFMs) based on three high-emissions Shared Socioeconomic Pathway SSP5-8.5 General Circulation Models (GCMs) under CMIP6^45^: MRI-ESM2.0, MIROC6, and IPSL-CM6A-LR. We defined two spatial agreement metrics: the Consensus Suitability Forecast (CSF), which includes areas classified as suitable by all three EFMs, and the General Suitability Forecast (GSF), encompassing areas considered suitable by at least one EFM. To estimate potential human exposure, we estimated the population living within climatically suitable areas using global demographic datasets^46,47^. A detailed comparison of EFMs and GCM-specific scenarios is provided in the **Materials and Methods section and Supplementary Results SI1**.

**Fig. 1:**
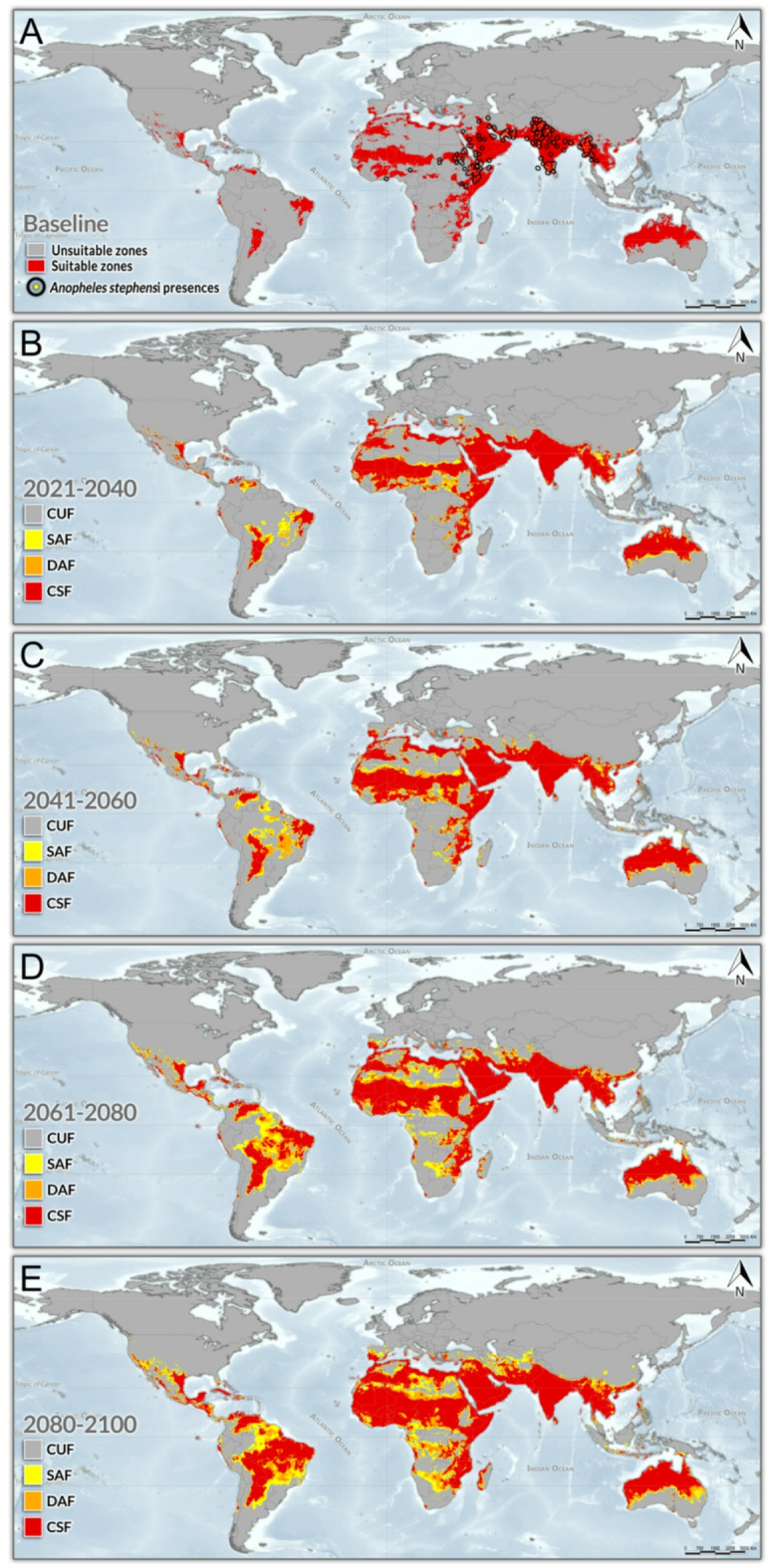
**Global Climate Suitability Scenarios for** ***Anopheles stephensi***. (**A**) Baseline climate conditions (1970–2000). Black rings indicate the empirical presences of *An. stephensi*. Projected climate change scenarios for (**B**) 2021–2040, (**C**) 2041–2060, (**D**) 2061–2080, and (E) 2081–2100. All scenarios are based on the latest Shared Socioeconomic Pathways SSP5-8.5 (CMIP6), and were projected using Global Circulation Models (GCMs) from MIROC, MRI, and IPSL (CMIP6, SSP5-8.5). Yellow areas represent suitability predicted by Ensemble Forecasting Models (EFMs) using the Global Climate Model (GCM) from a single institution (regardless of the institution), referred to as the Single Agreement Forecast (SAF). Orange areas indicate suitability based on EFMs using GCMs from two institutions (regardless of the institution), defined as the Double Agreement Forecast (DAF). Red areas denote shared zones across EFMs generated with GCMs from all three institutions, indicating total agreement in climatic suitability for *An*. *stephensi*, named as Consensus Suitability Forecast (CSF). The gray areas, referred to as the Consensus Unsuitability Forecast (CUF), represents climatically unsuitable areas across EFMs generated with GCMs from all three institutions. The General Suitability Forecast (GSF) is the combination of forecasts from all GCMs (CSF + SAF + DAF), regardless of the institution or the degree of overlap among forecasts. This figure was generated using the R programming platform (v. 4.3.1) and ArcGIS Pro software (v. 3.3).

**Fig. 2:**
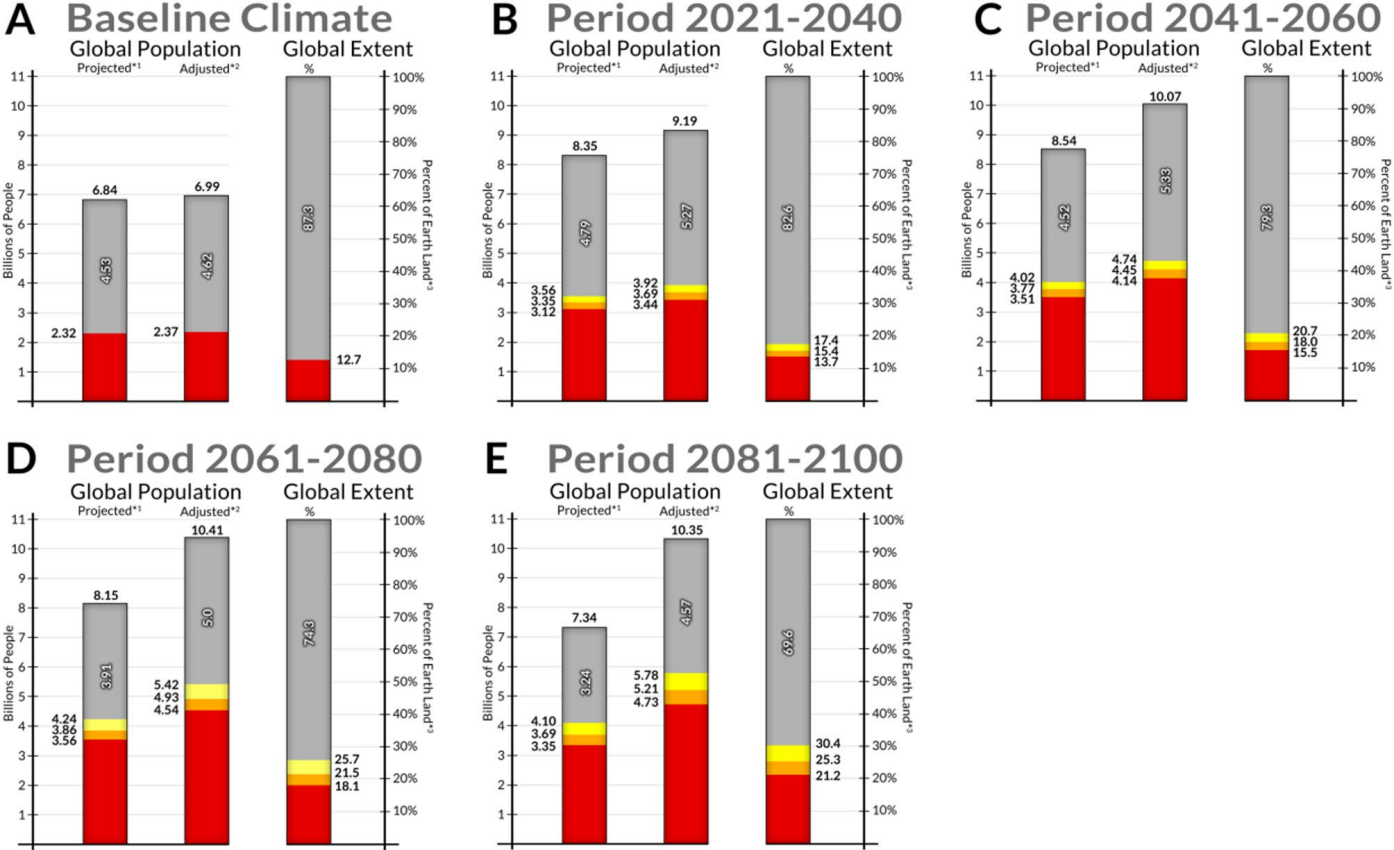
**Global population (in billions) and global extent (percentage of Earth’s dry land surface**,** excluding latitudes below S 59.25°) covered by climatically suitable areas for** ***An***. ***stephensi***. (**A**) At baseline, (**B**) 2021–2040, (**C**) 204,102,060, (**D**) 2061–2080, (**E**) 2081–2100.

Under the baseline climate scenario (1970–2000), almost 13% of the Earth’s dry land surface (excluding Antarctica), equivalent to over 17 million km², was climatically suitable for *An*. *stephensi* (Figs. 1A and 2A). This included highly populated countries with densely urbanized areas, such as China, India, Pakistan, and Nigeria. The suitable area encompassed a population of approximately 2.37 billion people^46^, representing about 34% of the world’s population (Fig. 2A). Climatically suitable areas for *An*. *stephensi* were projected to expand continuously in the subsequent decades, with an additional 2.36 billion (CSF) to 3.41 billion (GSF) people living in suitable areas from the baseline period to 2100, bringing the total global vulnerable population to between 4.73 billion (CSF) and 5.78 billion (GSF) by 2100 in the adjusted estimates (Figs. 1B-E and 2B-E).

Among the 256 countries considered in the analysis, 167 were found to have at least one cell (2.5 arc minutes or cells with around 21.5 km² at the equator) of climate suitability for *An. stephensi* by 2100 under either the CSF or GSF ensemble forecasts. Climatic suitability for *An. stephensi* was projected to potentially encompass all high malaria-burden countries in Africa by 2100, including Burkina Faso, Cameroon, the Democratic Republic of Congo, Ghana, Mali, Mozambique, Niger, Nigeria, Uganda, and Tanzania (Table 1**and Full dataset available in Supplementary Data SI3 - Dataset S1)**.

**Table 1:** Baseline and forecasted Climatic suitability for *Anopheles stephensi*, and demographic and malaria indicators for country groupings. Country specific information database can be accessed in **Dataset S1** in the **Supporting information (SI)**.

Indicator	Group 1	Group 2	Group 3	Group 4	Group 5
	(*n* = 14)	(*n* = 6)	(*n* = 51)	(*n* = 11)	(*n* = 85)
*Anopheles stephensi* presence	14 countries	6 countries	0	0	0
***Malaria***					
Malaria Cases 2022	92 813 464	0	155 669 308	37 803	0
Malaria Deaths 2022	245 070	0	363 090	0	0
***Population***					
Countries Population 2022 (thousands)	2 467 201	1 537 395	1 684 775	314 289	1 170 501
***Area***					
Countries Area km² (thousands)	13 581	12 346	35 581	3 055	32 668
Baseline Suitable % Coverage (average)	63.3	60.7	24.9	15.4	26.2
CSF % coverage by 2100 (average)	79.1	74.9	47.8	37.7	47.7
GSF% coverage by 2100 (average)	89	81	71.3	74.5	68.9
Baseline Suitable Area km² (thousands)	8 137	2 221	6 185	619	5 757
CSF Suitable Area km² 2100 (thousands)	10 311	3 000	14 748	1 245	9 010
GSF Suitable Area km² 2100 (thousands)	11 901	3 537	23 723	2 062	13 434

By 2100, 57 African countries are expected to exhibit climatic suitability for *An. stephensi*. Among these, 45 are currently affected by malaria, including seven that have already reported the presence of *An. stephensi*. Additionally, 12 of these countries are presently malaria-free. On average, the extent of suitability across African countries was predicted to increase by 22% (CSF) to 45% (GSF) compared to the baseline. The largest spatial expansions by 2100 were projected in Sudan, with an addition of 823,750 km² under the CSF (increase of 43.7% from baseline extent, covering 72.3% of country´s area) and 1,206,825 km² under the GSF (increase of 64%, covering 92% of country’s area), followed by Algeria (CSF: 723,805 km² or 30.4%, covering 57.1% ca.; to GSF: 1,285,019 km² or 54%, covering 80.7% ca.), and Libya (CSF: 515,759 km² or 29.3%, covering 43.2% ca.; to GSF: 1,221,098 km² or 69.4%, covering 83.3% ca.).

In Asia, *An. stephensi* is already present in 13 countries, including six that are currently malaria-free (Table 1**and Supplementary Data SI3 - Dataset S1)**. On average, climatically suitable areas in Asian countries were projected to expand by over 10% under the CSF and over 22% under the GSF from the baseline to 2100. The largest extent expansions by 2100 were predicted in Iran, with an additional 254,000 km² under the CSF (increase of 15.4% from baseline extent, covering 45.6% of the country’s area) to 725,000 km² under the GSF (increase of 44% from baseline extent, covering 74.2% of the country’s area), followed by China (CSF: 241,000 km² or 2.5%, covering 3.9% ca.; to GSF: 683,000 km² or 7.1%, covering 8.5% ca.) and Saudi Arabia (CSF: 370,000 km² or 17.2%, covering 99.5% ca.; to GSF: 380,000 km² or 17.7%, covering 100% ca.). By 2100, in addition to covering 14 Asian countries already severely affected by malaria, another 30 currently malaria-free countries were expected to exhibit climatic suitability for *An. stephensi*.

In Europe, the greatest increase in climatic suitability for *An. stephensi* was projected to occur in the Mediterranean region. Eight European countries were expected to experience a continuous increase in climatic suitability by 2100 (Table 1**and Supplementary Data SI3 - Dataset S1**). On average, the extent of suitability in these countries was predicted to grow by around 6% (CSF: covering on average 42% of the country’s area) to 16% (GSF: covering on average 53% of the country’s area) compared to the baseline (country’s area average: 36.4%). The countries with the largest expansions of climatic suitability area by 2100 were Spain (an addition of around 47,400 km² or 9.4%, covering 23.7% of the country’s area under CSF; to around 165,400 km² or 32.7%, covering 47% of the country’s area under GSF), Portugal (CSF: 13,000 km² or 14%, covering 61% ca.; to GSF: 43,500 km², or 47.2%, covering 94.2% ca.), and Greece (CSF: 16,700 km² or 12.6%, covering 29% ca.; to GSF: 34,000 km² or 25.8%, covering 94.2% ca.).

In the Americas, an even more notable increase in the area with climatic suitability for *An. stephensi* was detected, with an estimated average growth of around 35% (CSF: covering on average 48.6% of the country’s area) to 65% (GSF: covering on average 78.3% of the country’s area) from the baseline scenario (average 13.8%) to 2100 (Table 1**and Supplementary Data SI3 - Dataset S1**). This increase was detected in 49 American countries, including 16 that are malaria-endemic and one where malaria has been eliminated (Argentina). Additionally, 32 countries not reporting malaria were projected to exhibit climatic suitability by 2100. The countries with the largest expansions in climatically suitable areas by 2100 were Brazil (an addition of around 3,100,000 km² or 36.5%, covering 45.3% of the country’s area under CSF; to around 6,000,000 km² or 70.8%, covering 79.6% of the country’s area under GSF), Mexico (CSF: 374,0000 km² or 19%, covering 33.8% ca.; to GSF: 820,000 km² or 41.7%, covering 56.5% ca.), and Bolivia (CSF: 560,000 km² or 50.8%, covering 63.4% ca.; to GSF: 654,000 km² or 59.5%, covering 72.2% ca.).

In Oceania, Australia stood out for its already extensive baseline climatic suitability coverage, with more than 40% of the country’s area deemed suitable for *An. stephensi* (around 3,130,000 km²). This vast suitable area was projected to expand further by 9% (CSF: an addition of around 730,000 km², covering 49.8% of the country’s area) to 23% (GSF: 1,800,000 km², covering 63.7% ca.) by 2100 (Table 1**and Supplementary Data SI3 - Dataset S1**).

In addition to Australia, eight other countries in Oceania were projected to exhibit climatic suitability. On average, these countries were expected to experience an increase in suitability ranging from 10% (CSF: covering on average 14.6% of the country’s area) to 32% (GSF: covering on average 36.5% of the country’s area). Beyond Australia, Papua New Guinea (CSF: addition of around 13,000 km² or 2.8%; to GSF: addition of around 42,519 km² or 9.2%), and Fiji (CSF: 60 km² or 0.3%; to GSF: 3,000 km² or 16.2%) followed with the largest increases in spatial extent by 2100. Among the E-2025 countries^30^, only the Democratic People’s Republic of Korea, the Republic of Korea, and Vanuatu showed no climatic suitability for *An. stephensi* under any scenario.

From this analysis, five groups of countries were identified based on their climatic suitability for *An. stephensi* and their malaria epidemiology (Table 1). First, countries reporting the presence of *An. stephensi*, with malaria cases and associated deaths. This group included 14 countries with confirmed malaria cases and deaths in 2022 (Thailand and Iran did not report deaths)^30^ and the presence of *An. stephensi*. In Nigeria, the country with the highest malaria burden (both in cases and deaths), more than 36% of the territory was detected as climatically suitable for *An. stephensi* in the baseline scenario, a figure projected to increase significantly to 87% under CSF (an addition of around 50% or 465,000 km² from baseline) or to 98% under GSF (an addition of around 62% or 570,000 km² from baseline) by 2100. In addition to Nigeria (Deaths in 2022:189,321; Case Fatality Rate: 0.28), Kenya (D:11,788; CFR:0.35), Ghana (D:11,557; CFR:0.22), Ethiopia (D:10,570; CFR:0.21), Sudan (D:7,868; CFR:0.23), Somalia (D:2,507; CFR:0.24), and Yemen (D:2,222; CFR:0.25) present extensive areas of climatic suitability and established vector presence, making them highly vulnerable to *An. stephensi* expansion into urban regions **(**Table 1**and Supplementary Data SI3 - Dataset S1)**. These countries also bear the highest burden of malaria deaths and the highest mortality rates relative to their populations globally. Together, this group^1^ comprises about 2.5 billion people, with national populations ranging from more than 1.1 million in Djibouti to more than 1.4 billion in India.

The second group includes six countries that are certified malaria-free but where *An. stephensi* has been detected **(**Table 1**and Supplementary Data SI3 - Dataset S1)**. All have large areas of climatic suitability. In Saudi Arabia, about 82% of the country extent (~ 1,770,000 km²) is climatically suitable in the baseline scenario, and this is projected to cover to the entire country by 2100 in both ensembles, CSF and GSF. This is followed by Iraq (203,600 km² or 46% of the country’s area), China (130,000 km² or 1.4% ca.), the United Arab Emirates (84,000 km² or 100% ca.), Sri Lanka (23,000 km² or 34.3% ca.), and Qatar (12,000 km² or 100% ca.), which are the countries with the largest climatic suitability coverage at baseline scenario in this group. Home to nearly 1.5 billion people, much of the land area of these countries consists of deserts and mountains, resulting in high population concentrations in urban areas. The combination of high population density in urban zones with expanding *An. stephensi* suitability posed significant risks to their malaria-free status^30^.

The third group includes 51 countries with reported malaria cases and deaths but no recorded presence of *An. stephensi* **(**Table 1**and Supplementary Data SI3 - Dataset S1)**. Together, these countries were home to almost 1.7 billion people and reported about 156 million malaria cases and more than 363,000 deaths in 2022^30^. In Benin, where approximately 37,000 malaria cases per 100,000 people were reported in 2022, 58% of the territory was climatically suitable for *An. stephensi* in the baseline scenario and is projected to reach 100% by 2100 (CSF and GSF). The Democratic Republic of the Congo and Uganda, which had the highest number of malaria cases and related deaths in this group, had only about 6% and 7% of their areas suitable for *An. stephensi* in the baseline scenario. By 2100, however, this was projected to increase to 48% and 39%, respectively, according to the GSF forecast. By 2100, Burkina Faso and Mali were projected to have almost complete climatic suitability coverage under both the GSF and CSF forecasts, whereas Liberia, which was unsuitable for *An. stephensi* in the baseline, was projected to have 15% (CSF) to 74% (GSF) of its area suitable for the vector by 2100. At baseline, Bangladesh (96.3%), Burundi (83.9%) and Cambodia (83.4%) had the highest proportions of climatically suitable area covering. In terms of absolute extent, Mali, Brazil, and Niger each had about 740,000 km² of climatically suitable area, followed by Mauritania (510,000 km²) and Tanzania (440,000 km²). Brazil was the most populous and largest country in this group, with about 210 million people (87% living in urban areas), and a country’s area of about 8.5 million km²^48^. Brazil had the largest climatically suitable area for *An. stephensi* in the Americas (the fifth largest globally) and ranked first in the Americas in terms of malaria cases^30^.

In the fourth group, countries reported malaria cases in 2022, but no deaths and no evidence of *An. stephensi*, despite presenting areas climatically suitable for the vector in some of the periods considered **(**Table 1**and Supplementary Data SI3 - Dataset S1)**. In this group, Mexico, Vietnam, Nepal, Nicaragua, and Honduras had the greatest climatic suitability extents at baseline scenario. Vietnam, Nicaragua, and Honduras were projected to have more than 90% of their territory becoming climatically suitable for *An. stephensi* by 2100. Mexico stood out as the largest and most populous country in this group, with 130 million people living within its approximately 2 million km² territory. While only about 15% of its area was suitable in the baseline scenario, this was projected to increase to over 56% by 2100.

The fifth group included 85 countries that are certified malaria free and had not reported the presence of *An. stephensi* **(**Table 1**and Supplementary Data SI3 - Dataset S1)**. However, climatically suitable areas for the vector were detected. Specifically, ten countries (Kuwait, Palestine, Northern Cyprus, Bahrain, Curaçao, Malta, Akrotiri and Dhekelia, Aruba, Paracel Islands, and Gibraltar) were detected to reach 100% suitability coverage by 2100. In Argentina, 10% of territory is climatically suitable in the baseline scenario, projected to increase to between 18% (CSF) and 30% (GSF) by 2100. In terms of absolute suitable area at baseline scenario, Australia (3,130,000 km² or 40.4% of the country’s area), Algeria (637,000 km² or 26.7% ca.), Oman (300,000 km² or 96.9% ca.), Argentina (286,000 km² or 10.3% ca.), and Morocco (250,000 km² or 56% ca.) ranked as the largest.

Lastly, to enhance the biological relevance of model outputs, we performed a post hoc climatic profiling of areas classified as suitable in the ensemble baseline scenario. Climatically suitable regions were characterized by mean annual temperatures predominantly between 18 °C and 32 °C, aligning with known physiological thresholds for mosquito and parasite development^38,40^. The vast majority of suitable cells also coincided with regions where mean relative humidity surpassed 60%, a key determinant of adult mosquito survival and transmission probability^39,41^. This climatic envelope supports the plausibility of suitability outputs, especially across newly projected areas of potential invasion. The complete results can be accessed in Supplementary Results SI1.

## Discussion

An advanced modeling framework was developed to estimate current and projected areas with climatic suitability for *An. stephensi*, as well as the population living in those areas. Our results show that 76 malaria-endemic countries have regions climatically suitable for *An. stephensi*, yet only 14 have confirmed its presence. Furthermore, six malaria-free countries report both the presence of *An. stephensi* and suitable climatic conditions. By 2100, 167 countries are projected to exhibit climatic suitability for *An. stephensi*. Currently, approximately 30% of the total area of these countries is already characterized by suitable conditions (12.7% of global dry surface), which could increase to between approximately 50% (21.2% of global dry surface) and 72% (30.4% of global dry surface) under the CSF and GSF scenarios, respectively. This is expected to elevate risks significantly in malaria-endemic regions and pose a major threat to malaria-free countries, potentially exposing 5.8 billion people of an estimated global population of ~ 10 billion. Thus, the ability of *An. stephensi*, particularly its Type and Intermediate forms, to thrive in urban environments threatens immunologically naive populations in regions with historically low malaria transmission^49^. The 20th century invasion of *An. arabiensis* in northeastern Brazil^50^ and its recent detection in Côte d’Ivoire^51^ exemplify the dangers associated with human-mediated dispersal of malaria vectors. These cases emphasize the need for effective monitoring systems, particularly at maritime ports, airports and along trade routes connected to regions where *An. stephensi* has been reported to curb the expansion of this invasive species.

Results of the climatic suitability modeling demonstrate that both malaria-affected and several malaria-free countries already possess areas with climatic conditions suitable for *An. stephensi* across multiple scenarios. Projections indicate a substantial expansion of *An. stephensi* range in the coming decades, with climatic suitability encompassing 50% of the global population by 2072 and nearly 5.8 billion people at risk of acquiring malaria by 2100. Currently, only 14 countries report *An. stephensi* as either an invasive or endemic species. This presents a critical opportunity for early detection of potential invasions, establishment of the species, and the implementation of proactive policies to limit its spread and curb the expansion of malaria occurrence. Invasive species often fail to establish in new areas when propagule pressure is insufficient or when the probabilities of introduction and establishment are low^52,53^. A similar process has been observed in river-dependent anophelines of the Amazon Basin, where dispersal is heavily influenced by the direction of river flow or prevailing winds^54^.

The use of calibrated (comparable), spatially explicit data in modeling highlights the critical importance of methodological rigor to guide policy development and design effective intervention strategies. A recent study^55^, employing predictive models based on current and future climate settings, demonstrated that climate change is likely to expand suitable urban habitat for *An. stephensi* across Africa, Asia, and other regions. Additionally, Ryan et al.. (2023; [44]) identified a poleward expansion of potential habitats under future climate scenarios, noting that over 7 billion people are already at risk, with exposure projected to increase.

The central role of predictive modeling in understanding and preparing for the invasive global spread of *An. stephensi* is broadly applicable to other vector species. By employing multiple, most applied (based on Scopus citation) and updated GCMs (CMIP6) the present study projected future climatic suitability for *An. stephensi*across space and time. Among these general circulation models, MIROC-GCM (MIROC6^56^;) predicted less favorable conditions for the vector, a less concerning scenario for public health. Conversely, IPSL-GCM (IPSL-CM6A-LR^57^;) and MRI-GCM (MRI-ESM2-0^58^;) projections suggested more disconcerting outcomes, with greater areas of climatic suitability. Variability among models is commonplace, requiring the need to integrate GCMs from multiple institutions and interpreting results through diverse perspectives (e.g., CSF and GSF).

The shared ecology of *Aedes aegypti* and *An. stephensi*, both of which breed in water-filled artificial containers in urban areas, positions them as highly capable of exerting substantial propagule pressure and expanding globally. Effective mitigation of this pressure requires proactive vector control and surveillance measures before these species become established in a new environment. Historical precedent, such as the successful efforts of Fred Soper and his team in preventing the establishment of *An*. *arabiensis* in northeastern Brazil^59^, illustrates the importance of early intervention. The introduction of *An. arabiensis* to Brazil in the 1930s highlights the risks associated with human-mediated mosquito dispersal and the role of maritime transport^50,51^. This spread was likely facilitated by water-filled maritime cargo containers carrying mosquito eggs or larvae, prompting public health responses such as habitat elimination, drainage initiatives, and insecticide applications^51,59^. Similarly, the global transport of water-holding containers significantly enhances *An. stephensi* potential to invade and be established in urban settings^8,24,29^. Unlike *An. arabiensis*, which is generally associated with rural or agricultural areas, *An. stephensi* thrives in urban and human-modified settings, showcasing a greater capacity for long-distance dispersal than most other malaria vectors. This adaptability, certainly, makes controlling and eliminating *An. stephensi* more challenging than the elimination of *An. arabiensis* from northeast Brazil. The successful elimination of *An. arabiensis* in Brazil was largely due to the implementation of a strict vector control program grounded in field evidence.

Ecological parallels between *An. stephensi* and *Aedes aegypti* (urban vector of Dengue, Zika, and Chikungunya viruses), such as similar larval habitats and urban environmental adaptability exacerbate public health threats^8,60^. The built-environment coexistence of these species is likely to worsen the burden of mosquito-borne diseases, especially given the inconsistent success of *Aedes* interventions^61^. This is particularly noticeable where rapid urbanization has outpaced the development of critical infrastructure, leaving millions without access to clean water, adequate sanitation, or effective waste management^62–64^. Unplanned expansion can lead to environmental degradation, increasing vulnerability to climate-related disasters^65,66^.

The climatic suitability range identified in our projections - predominantly 18–32 °C with elevated relative humidity - aligns closely with well-established biological thresholds for *An. stephensi* and malaria parasites in the literature. Experimental and field-based studies have demonstrated that temperatures below 18 °C sharply reduce vector survival during the extrinsic incubation period, while temperatures above 32 °C increase mosquito mortality, even if parasite development accelerates^38,39^. Additionally, relative humidity above 60% is crucial to ensure adult longevity sufficient to complete sporogony^41^. Our modeling outputs corroborate these ecological constraints, reinforcing their relevance for public health planning and vector control strategies. Furthermore, operational control of *An. stephensi* may be severely constrained by widespread pyrethroid resistance, as recently documented in Ethiopia across all major pyrethroid compounds^67^. This further underscores the urgency of early detection, resistance monitoring, and the implementation of integrated vector management in newly affected regions, since the spread of resistant *An. stephensi* may unintentionally facilitate its rapid expansion.

Innovative tools such as environmental DNA detection (eDNA) have the potential to greatly improve early intervention and control efforts by enabling simultaneous identification of multiple species, even at low larval densities^68^. The reduction of mosquito habitats in peri-urban areas could be achieved by the integration of agricultural policies with real-time weather and land-use data to optimize irrigation practices^69^. Targeted surveillance strategies in urban and coastal regions are essential for accurately mapping *An. stephensi* distribution, understanding its bionomics, and assessing its potential for disease transmission in previously non-endemic areas^20^. This study offers valuable insights for policymakers by elucidating the role of climate change in shaping the climatic suitability of *An. stephensi*.

While our models focus on climatic suitability for *An. stephensi*, actual malaria transmission dynamics also depend on environmental thresholds required for the development of *Plasmodium falciparum* and *P. vivax* within the mosquito. Warmer temperatures in newly suitable areas may shorten the extrinsic incubation period (EIP), potentially increasing transmission efficiency. However, transmission could remain limited in some regions if relative humidity remains too low for adult vector survival, or if factors such as low human population density, poor vector–host contact, or strong health systems reduce the likelihood of sustained transmission. In this context, our projections should be interpreted as potential zones of vector–parasite compatibility, rather than deterministic maps of disease expansion. Future work integrating Plasmodium-specific thresholds and infection dynamics will be essential to refine malaria risk forecasting under climate change.

In response to the global expansion of *An. stephensi*, several innovative vector control strategies are currently under development or implementation. These include the *Sterile Insect Technique* (SIT), involving the large-scale release of sterilized males to suppress wild populations; *Wolbachia*-based biocontrol, where endosymbiotic bacteria are used to reduce pathogen transmission or impair mosquito fitness; and targeted larval source management, especially in urban water storage systems that serve as primary breeding habitats for *An. stephensi* (and *Aedes aegypti*). When integrated with early warning systems and geospatial suitability forecasts - such as those provided by this study - these approaches offer valuable tools for proactive containment and mitigation of *An. stephensi* invasions under both current and future climate conditions.

## Methods

### Modeling framework

To evaluate the global climatic suitability and the impact of climate change on *An*. *stephensi*, a robust multi-modeling framework was developed. This approach integrated Geographic Information System tools (ArcGIS Pro, v.3.3), the R programming platform (v.4.3.1), and the *Biomod2* package (v. 4.2.2; [70]). Eight algorithms were employed: Flexible Discriminant Analysis, Generalized Linear Model, Generalized Additive Model, Random Forest, Maxent - Maximum Entropy, MaxNet - Maximum Entropy, Extreme Gradient Boosting, and Multiple Adaptive Regression Splines (details on the modeling framework are available in the **Supplementary Methods SI2**).

## Climate scenarios

The framework utilized high-resolution global climate data from WorldClim v2.1^71^ for baseline conditions (1970–2000) and future projections (2021–2100) under the SSP5-8.5 scenario, leveraging data from the CMIP6 initiative. Nineteen bioclimatic variables were used representing baseline climatic conditions. To ensure the robustness of predictors, variables were filtered for collinearity using a Variance Inflation Factor test (*usdm* R package v.2.1–7;^72^). Future projections were based on three GCMs: IPSL-GCM^55^, MIROC6-GCM^51^, and MRI-GCM^53^. These GCMs were selected based on their citation frequency in Scopus (variables list, selection criteria and sources were described in **Supplementary Methods SI2**).

## Data collection and filtering

Presence records for *An. stephensi* (*N* = 756) were compiled from Malaria Atlas Project^73^, GBIF (DOI:10.15468/DL.VHGR3P), and gathered from scientific literature^12,22,23^. These records underwent rigorous filtering to exclude duplicates, improbable locations, and non-relevant sites reports (e.g., forested areas without human habitation). Pseudo-absence data (at ten times the number of presences per modeling round) were selected from locations where other *Anopheles* species were recorded and from worldwide randomly selected points, with constraints to avoid overlap or proximity to presence records. To minimize spatial clustering and enhance generalization, multi-blocked data partitioning was employed. The *spThin* R package (v.0.2.0;)^74^ was used to create randomized subsets, while *Biomod2* embedded partitioning allocated 70% of presence data for model calibration and 30% for testing predictive quality^70^.

### Models selection and ensemble forecasts

Baseline models were evaluated using the True Skill Statistic (TSS)^75^, retaining only those with TSS ≥ 0.75 (*N* = 112) for future scenario projections. These models were then ensembled using the Committee Averaging method within the *Biomod2* package^70^. Two sequential batches of Ensemble Forecast Models (EFMs) were generated **(see Supplementary Information SI2 and SI1 respectivelly for details on methods and results comparisons among GCMs from Institutions)**. The first batch combined models by algorithm and periods (2021–2040, 2041–2060, 2061–2080, 2081–2100), generating institution-specific EFMs. The second batch integrated the EFMs from the first batch across the three institutions. This process resulted in the following forecast categories: **Consensus Suitability Forecast (CSF)** - areas consistently predicted as suitable by all EFMs across all future periods. This class represents full spatial overlap and unanimous agreement among EFMs based on GCMs from the three institutions; **Consensus Unsuitability Forecast (CUF)** - areas consistently predicted as *unsuitable* by all EFMs, integrating projections from all GCMs across the three institutions. This class denotes complete spatial and predictive agreement on climatic unsuitability; **Single Agreement Forecast (SAF)** - Areas predicted as suitable by EFMs from only one institution, irrespective of which institution; - Double Agreement Forecast (DAF) - areas predicted as suitable by EFMs from any two institutions, regardless of the specific institutions. Note that SAF and DAF classes were calculated and mapped, but not analyzed separately, both were integrated in the General Suitability Forecast; **General Suitability Forecast (GSF)** - areas identified as suitable by at least one EFM using GCMs from any institution. GSF represents the union of SAF, DAF, and CSF, thus delineating the most inclusive and spatially comprehensive extent of predicted climatic suitability for *An. stephensi*.

## Demographic accounting

To estimate the human populations exposed to *An. stephensi* under different climatic suitability scenarios, geospatial population data from NASA-SEDAC^47^ and the United Nations^46^ were integrated with suitability projections. Official malaria and demographic data (30; 46) were cross-referenced with modeling results, alongside demographic projections for future periods under SSP5-8.5, to identify and correlate vulnerable countries. A series of refinements were implemented, adhering to the most thoroughly tested protocols, to establish a robust and reliable framework. Comprehensive details on data sources, inputs, parameters, and outputs are provided in the **Supplementary Methods SI2.**

## Supplementary Information

Below is the link to the electronic supplementary material.

Supplementary material 1 (DOCX 4001.3 kb)

Supplementary material 2 (PNG 327.1 kb)

Supplementary material 3 (PNG 266.3 kb)

Supplementary material 4 (PNG 1001.9 kb)

Supplementary material 5 (PNG 1342.5 kb)

Supplementary material 6 (PNG 3338.9 kb)

Supplementary material 7 (PNG 2243.3 kb)

Supplementary material 8 (PNG 2833.4 kb)

Supplementary material 9 (XLSX 17.1 kb)

Supplementary material 10 (PNG 86.3 kb)

## Data Availability

All datasets can be downloaded from: A. Acosta, Future Global Distribution and Climatic Suitability of Anopheles stephensi. Zenodo. https://doi.org/10.5281/zenodo.15558088.
