# Supplementary material for: Future global distribution and climatic suitability of Anopheles stephensi
Source: Sci Rep. 2025 Jul 1;15:22268. doi: 10.1038/s41598-025-07653-8 (PMC12215484; doi:10.1038/s41598-025-07653-8)
Supplement: Supplementary file 1 — Supplementary material 1 (DOCX 4001.3 kb) [file 41598_2025_7653_MOESM1_ESM.docx]

**Scientific Reports**

**Supplementary information for “Future Global Distribution and Climatic Suitability of *Anopheles stephensi*”**

**SI1) Supplementary Results**

*Comparing Scenarios among GCMs from Institutions*

The results of climate modeling conducted in this study revealed substantial geospatial differences in climate suitability predictions for *An. stephensi* among EFMs generated with variables from the GCMs across three institutions, all based on the SSP5-8.5 scenario for the same periods. The EFMs derived from the IPSL-GCM projected the most concerning scenarios, indicating the largest global climate suitability for *An. stephensi* (**Figs. S1A and S1B**). According to models generated using the IPSL-GCM, by 2100, Earth's climate was expected to increase the suitable range for *An. stephensi* by 17%, reaching nearly 30% global coverage (**Fig. S1A**), an increase of almost 134% from the baseline (**Fig. S1B**). Conversely, but still worrisome, the EFMs based on MIROC-GCM and MRI-GCM projected less favorable future climates for the invasive species, predicting growth to about 10% and 12%, respectively, reaching nearly 23% (around 91% increase) and 24% (around 80% increase) of global coverage (**Figs. S1A and S1B**). Although the EFM from MIROC -GCM projected a lower expansion in future climate suitability compared to MRI-GCM, both exhibited similar trends in progressive suitability growth over time. MRI-GCM consistently projected slightly higher suitability than MIROC-GCM across each future period (**Figs. S1A and S1B**). In the context of global *An. stephensi* expansion and climate change, projections from MIROC-GCM were relatively less threatening to human health compared to the other EFMs.

Considering the comprehensive GSF ensemble forecast, which integrated suitability predictions from all three institutional GCMs regardless of prediction overlap levels or originating institutions (combining predictions from SAF, DAF, and CSF; SI **Figs. S2A and S2B**), the outlook appeared as dramatic as the most pessimistic perspective observed in the IPSL-GCM (**Figs. S1A and S1B**). A linear estimate considering the GSF indicates that by 2072, 50% of the global population - approximately 10 billion people - will reside in climatically suitable zones for *An. stephensi* (**Figs. S2A and S2B**). Alternatively, the CSF, which accounted for the complete predictive agreement among EFMs across GCMs while excluding individual EFM-GCM prediction divergences, offered a more cautious and less pessimistic assessment of the geographical suitability range of *An. stephensi*.

Both the GSF and CSF ensembles provided valuable insights to support decision-making, spatiotemporal prioritization for monitoring, and the planning of preventive measures and interventions across various contexts and demands. The GSF ensemble appeared particularly suited for prioritizing areas for large-scale, long-term monitoring, planning preventive measures, and conducting awareness and prevention campaigns, as it incorporated the combined strengths of explanatory variables from all institutions to generate the EFMs. Its broader scope, which equally weighted zones of prediction convergence and divergence among GCMs, reduced the likelihood of omission errors when selecting priority areas for intervention. Although it was not possible to determine which institution's predictions most robustly captured future climate conditions and thus contributed the most accurate suitability models, including suitability predictions from all GCMs substantially increased the likelihood of covering the zones most accurately predicted as suitable for one or more institutions. Conversely, the CSF ensemble, by focusing exclusively on suitable regions consistently and equally predicted by all EFMs (using variables from all GCMs and institutions), defined a more spatially restricted area but with a higher confidence level. This area represented zones of maximum agreement or overlap in suitability prediction, excluding marginal areas with conflicting predictions where intrinsic error was likely higher. Prioritizing this extent is optimal for maximizing the chances of detecting and observing *An. stephensi* in the field. The CSF proved particularly useful for prioritizing emergency control and impact mitigation actions, especially when resources allocated to vector control are limited. Its narrower spatial extent, compared to the GSF, enabled a concentrated focus on areas with the highest statistical likelihood of suitable climate conditions for the vector.

**
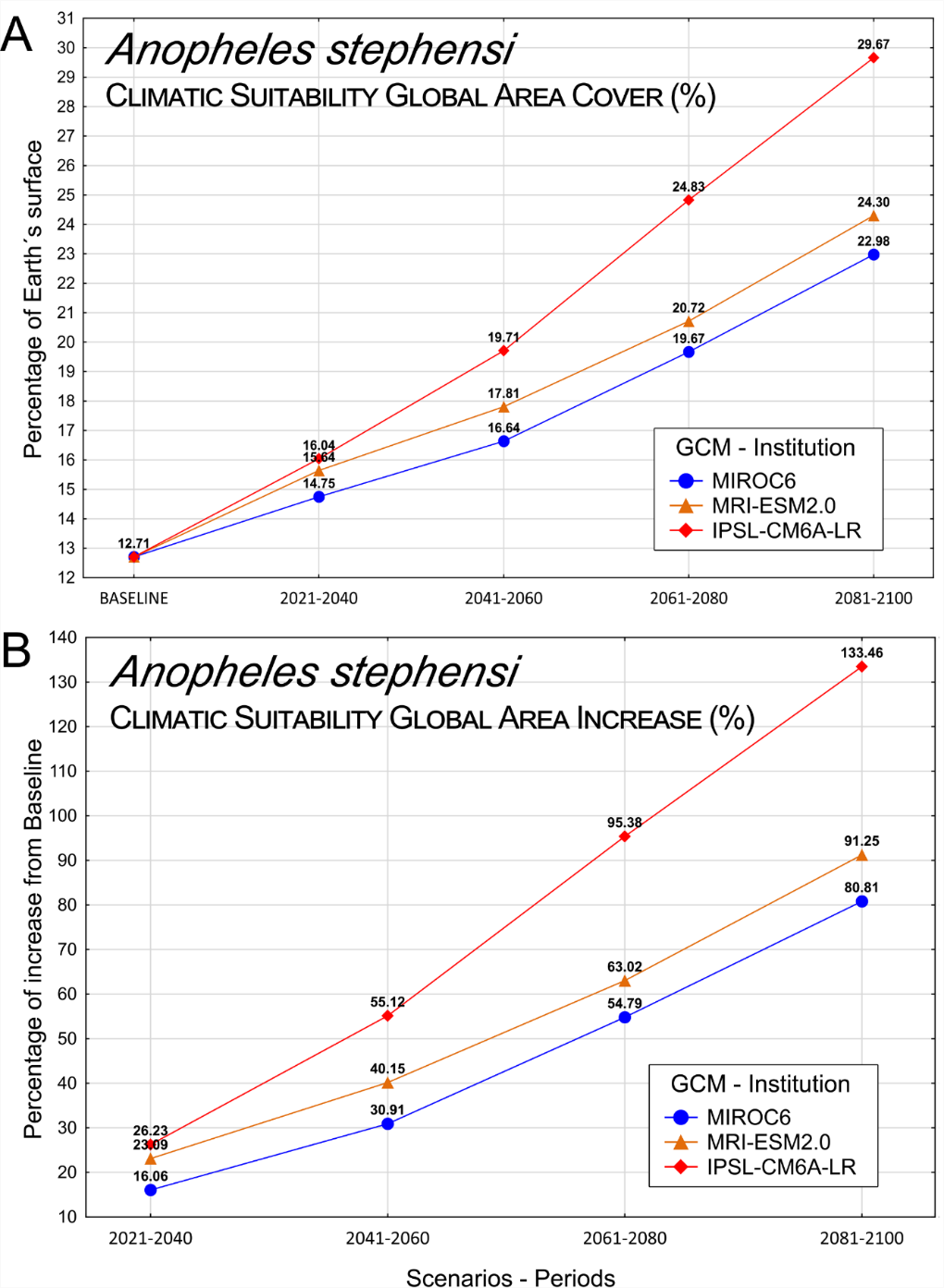
**

**Figure S1.** Percentage of global area climatically suitable for *An. stephensi* by GCM institution. (A) Extent (%) of suitable areas from the baseline period to each respective future period. (B) Increase (%) of suitable areas relative to the baseline period for each future period.


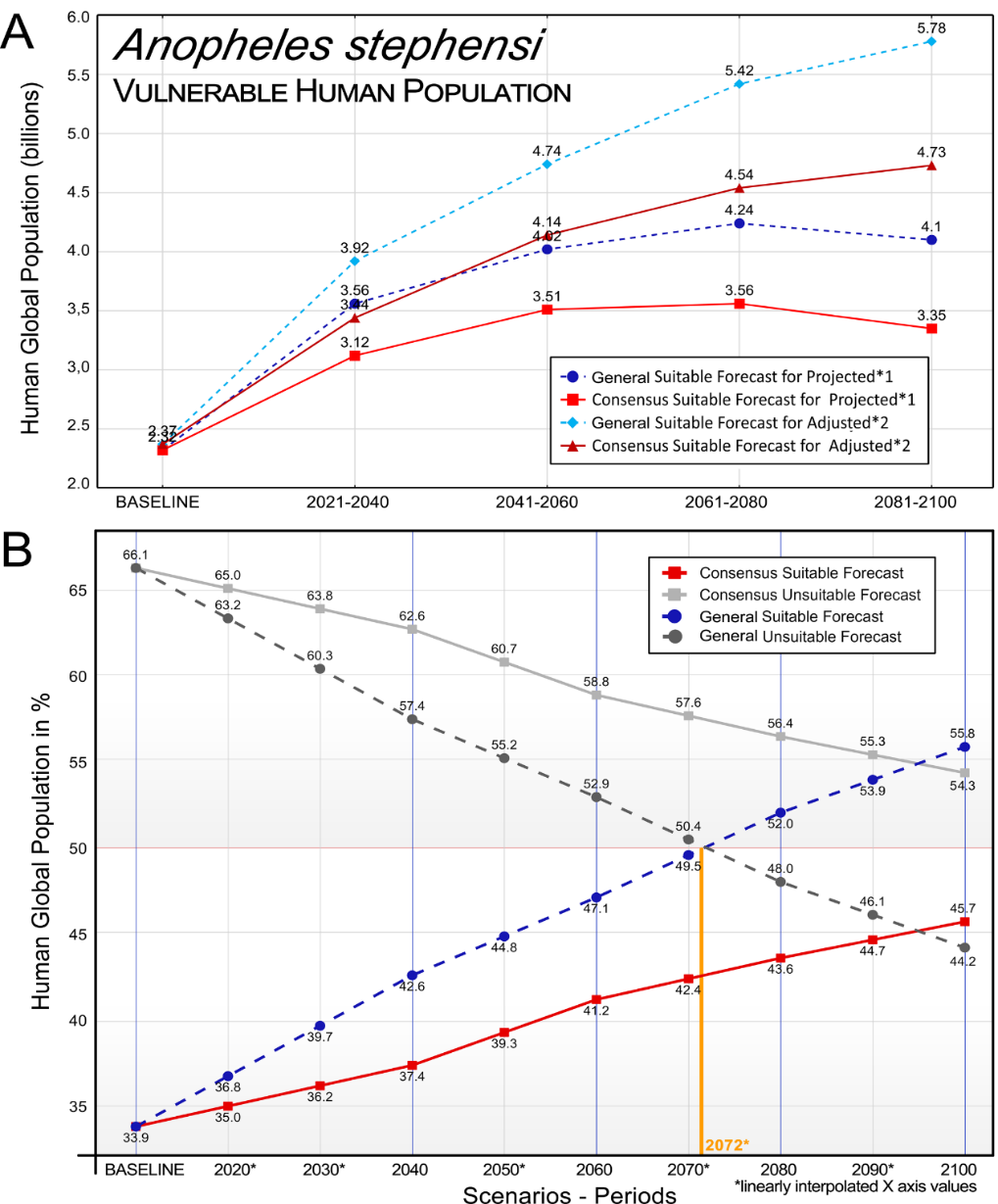


**Figure S2.** Global population (A) in billions and (B) the percentage of the population residing in climatically suitable areas for *An. stephensi* per period. The solid lines display CSF and the dashed lines the GSF, both generated using GCMs from MIROC, MRI, and IPSL. In (A), lines are representing demographic projections for future periods: the Projected line (*1) incorporates spatially demographic data from NASA-SEDAC (1), while the Adjusted line (*2) is calibrated using data from the United Nations (2). Figure B shows the percentages of the human population covered by climatically suitable areas for each decade. The asterisk-marked decades utilize linear interpolation for decades without data and to identify the inversion point of the GSF, which occurs in 2072 (orange line). At this point, over half of the human population will be exposed to vulnerable zones.

*Variable Importance and Environmental Ranges of Predictors*

The variable importance analysis revealed a clear hierarchy of predictor influence across four major environmental domains: temperature, rainfall, relative humidity, and altitude. The classification plot (**Fig. S3**) shows that temperature-related variables dominated in explanatory power, followed by precipitation seasonality.


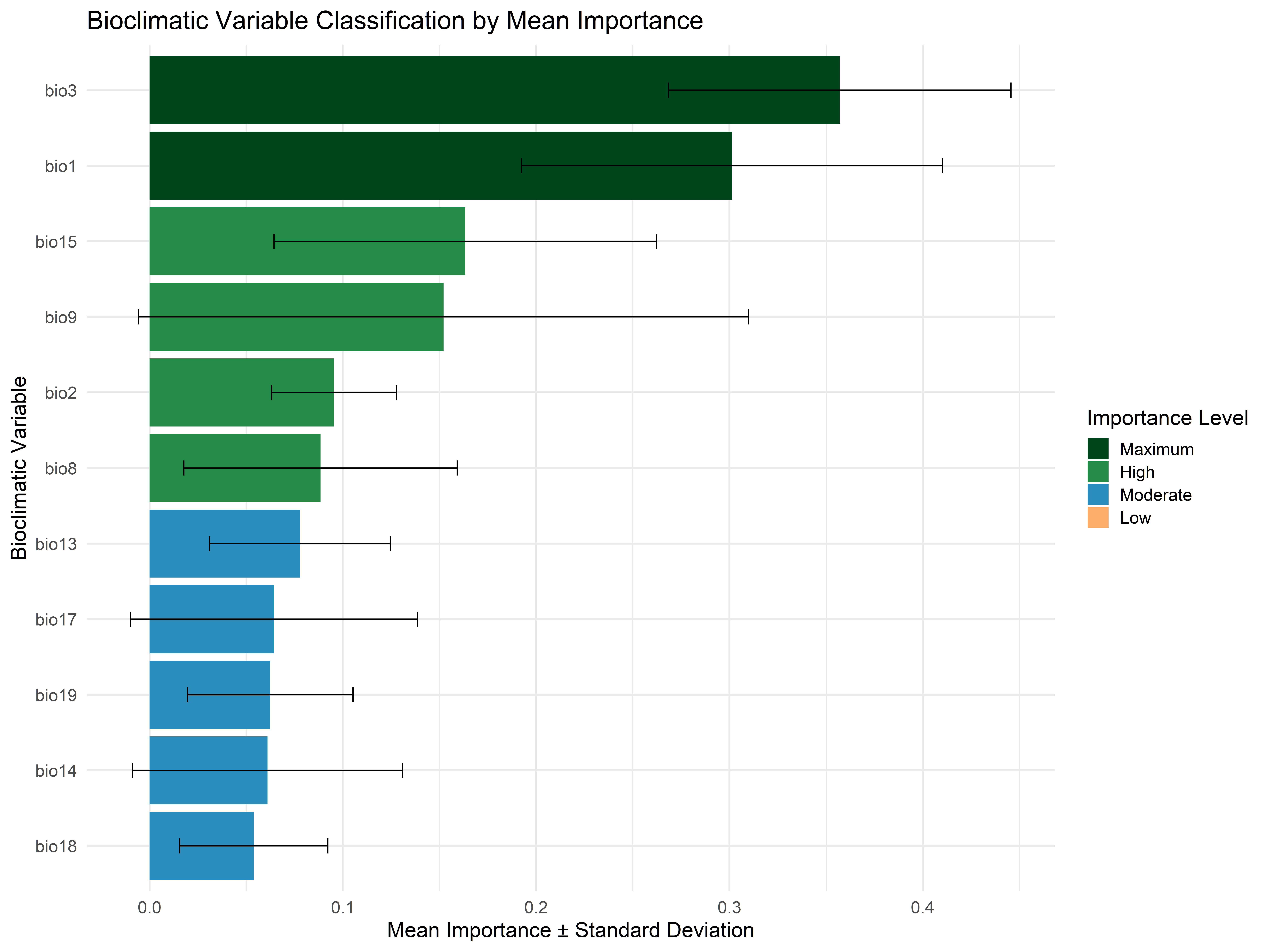


**Figure S3.** Classification of Bioclimatic Variables by Mean Importance. Mean variable importance scores ± standard deviation extracted from 112 validated ensemble models (TSS ≥ 0.75). Variables are grouped by quartile thresholds into four importance levels: Maximum (dark green), High (green), Moderate (blue), and Low (orange). The “Low” category does not appear in the graph, as no variable was classified within this range of relevance.

Rainfall was incorporated into the modeling through several bioclimatic variables. Overall, rainfall-related variables had lower average importance than temperature-related variables but remain ecologically significant. Among these, BIO15 (Precipitation Seasonality) was consistently classified as “High” and exhibited a strong explanatory signal. Suitable zones had BIO15 values (coefficient of variation) between 27.1 and 183.9 (median = 95), indicating the species can tolerate pronounced intra-annual variation in rainfall. This aligns with the mosquito’s behavior in breeding in transient water bodies during rainy pulses and its resilience in intermittently dry zones (3).

Other rainfall variables - BIO13 (Wettest Month), BIO14 (Driest Month), BIO17 (Driest Quarter), BIO18 (Warmest Quarter), and BIO19 (Coldest Quarter) - were ranked as “Moderate” in importance. Boxplot distributions show that even in the driest quarter (BIO17), suitable habitats can occur with only 2–57 mm of rainfall (median = 10 mm), suggesting the species can persist with minimal surface water availability, potentially relying on manmade containers. This is corroborated by reports of larval development in overhead tanks, barrels, and construction pits in water-stressed urban areas across India, Ethiopia, and Sudan (4).

Temperature was the primary climatic driver of predicted suitability for *An. stephensi.* BIO1 (Annual Mean Temperature) and BIO3 (Isothermality) were consistently ranked with “Maximum” importance. The thermal ranges in suitable areas (Fig. S4) show BIO1 values between 17.8°C and 31.2°C, with a median of 25.7°C, consistent with the vector’s established tolerance to warm tropical and subtropical climates. BIO3, which captures the ratio between diurnal and annual temperature variation, ranged from 24.9% to 79.8% (median = 50.4%), suggesting that thermal stability plays a strong role in habitat affinities. These findings are consistent with studies showing that *An. stephensi* thrives under relatively stable thermal conditions, both in native zones and in expanding urban environments where microclimate buffering reduces extreme fluctuations (5).

Two additional temperature variables were ranked with “High” importance: BIO9 (Mean Temperature of the Driest Quarter) and BIO2 (Mean Diurnal Range). Their ranges (BIO9: 11.7–36.0°C, median 24.1°C; BIO2: 5.8–18.2°C, median 13.0°C) highlight the species’ tolerance to high temperatures during dry seasons and to moderate intraday variability, a trait associated with its ability to persist in peri-urban water containers and ephemeral breeding sites (6). These thermal profiles reinforce that *An. stephensi* can exploit microhabitats with limited thermal buffering, a characteristic linked to increased urban malaria risk.


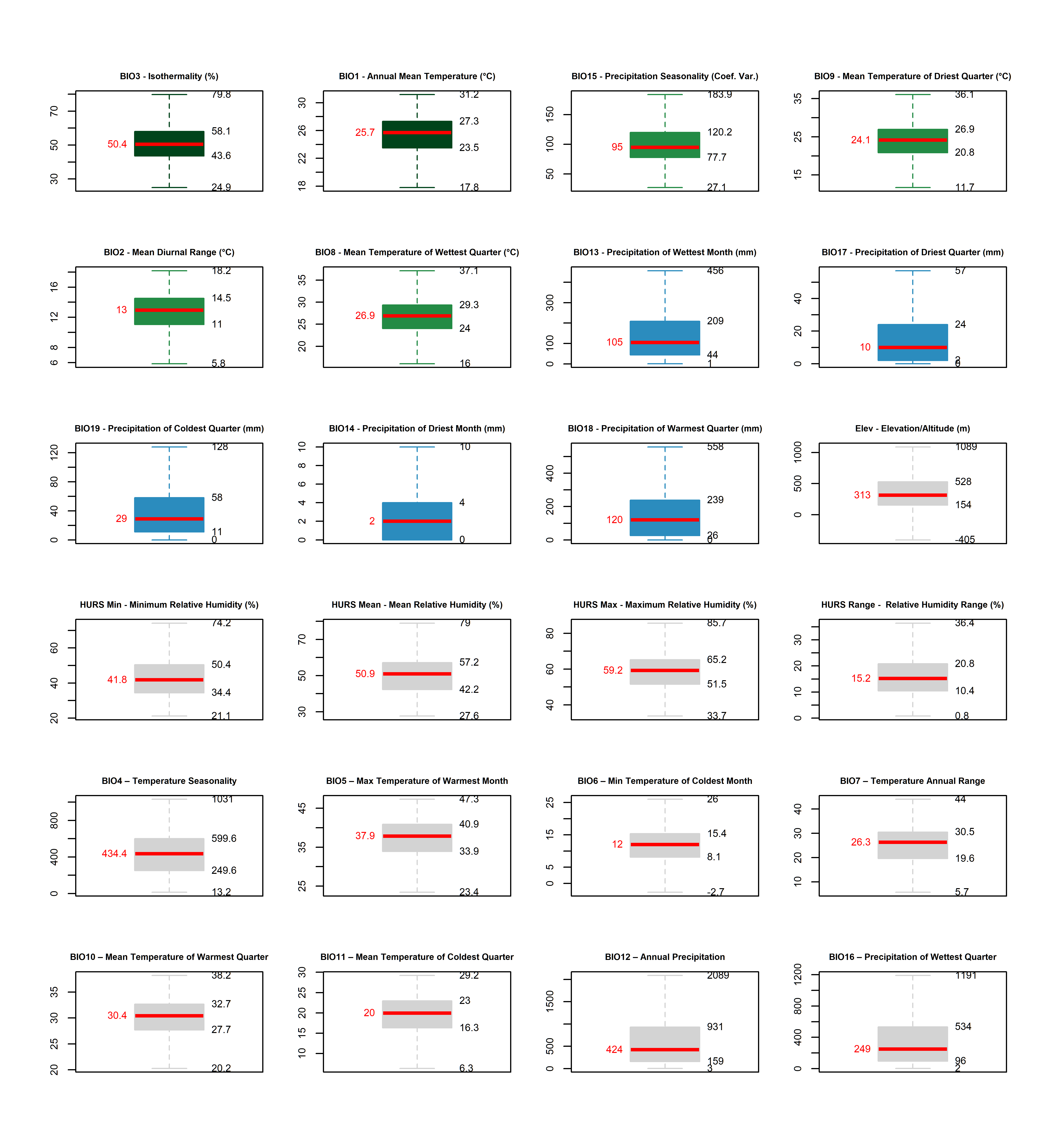


**Figure S4.** Boxplots of Environmental Variables in Suitable Areas. Distributions of 19 bioclimatic variables, elevation (7), and relative humidity metrics (8) across grid cells predicted as suitable for *An. stephensi* under baseline climate conditions. Each boxplot displays minimum, first quartile (Q1), median (red line), third quartile (Q3), and maximum values; outliers were removed prior to plotting. Boxplots are colored according to four importance levels: Maximum (dark green), High (green), and Moderate (blue). Gray boxplots represent variables that were either excluded (after VIF test) or not included (elevation and humidity variables) in the final modeling procedure.

Relative humidity, although not included in the modeling procedure due to the absence of sources providing projections for future climate scenarios (SSP-CMIP6), was analyzed post hoc by extracting baseline variable ranges (8) in baseline suitable areas to provide insights into the species’ ecological tolerance to atmospheric moisture.

Minimum humidity (hurs_min) ranged from 21.1% to 74.2% (median = 41.8%), while mean humidity (hurs_mean) ranged from 27.6% to 70.9% (median = 50.9%). Maximum humidity (hurs_max) extended from 33.7% to 85.7% (median = 59.2%), and relative humidity range (hurs_range) spanned from 0.8% to 30.4% (median = 15.2%). These values collectively indicate that *An. stephensi* can survive and reproduce under relatively dry atmospheric conditions, particularly in environments where indoor microclimates or anthropogenic water sources buffer desiccation risks. Although humidity variables were not considered in the global models, they remain ecologically relevant, especially for adult survival and egg viability in arid and semi-arid regions, where desiccation stress may constrain invasive expansion fronts (9).

Altitude, represented by elevation (7), was also not considered in the modeling due to its high collinearity with multiple bioclimatic variables. Nonetheless, it was analyzed post hoc by extracting its values in areas classified as suitable under baseline conditions. Elevation in these zones exhibited a wide empirical range (−405 to 1,089 m, median = 313 m), reflecting the species’ substantial vertical plasticity and strong association with urban environments, where altitude itself imposes minimal constraint compared to microclimatic and anthropogenic factors. Documented occurrences in coastal, lowland, and mid-elevation cities such as Khartoum, Addis Ababa, and Delhi further support the conclusion that altitude is a poor standalone predictor of suitability, unless tightly coupled with temperature gradients (10).

In summary, the spatial range of *An. stephensi* is primarily shaped by temperature regime and seasonal rainfall variability. While relative humidity and elevation play lesser roles in global-scale models, they likely interact with local land use, human behavior, and microclimatic effects to shape invasion potential. The ranges identified here provide ecologically coherent thresholds for forecasting the expansion of this vector, prioritizing entomological surveillance, and implementing targeted interventions in vulnerable regions.

**SI2) Supplementary Methods**

*Multi-Modeling Framework*

To assess the global climate suitability of *An. stephensi* across spatial and temporal dimensions, we developed a climatic suitability framework. This method integrates environmental factors with species distribution data to generate near-current and projected spatially explicit suitability models. The multiple models and spatially explicit projections were developed by integrating Geographic Information Systems (1), R programming platform (2), and the species distribution multi-modeling tool *Biomod2* (version 4.2.2; [11,12]) using eight algorithms:

FDA: Flexible Discriminant Analysis (version 0.5-5; [13]) is a non-parametric extension of Linear Discriminant Analysis (LDA), designed to model complex, non-linear relationships between predictor variables and categorical outcomes. Unlike LDA, which assumes linear class boundaries and equal covariance matrices, FDA relaxes these constraints by integrating optimal scoring with flexible regression models. The method operates in three main steps: (i) transformation of categorical responses into continuous scores via optimal scoring; (ii) fitting of a non-parametric regression model - typically based on multivariate adaptive regression splines (MARS) - to capture non-linearities and interactions; and (iii) application of discriminant analysis on the fitted values to identify axes that best separate classes. In this study, FDA was applied through biomod2 with default parameters. By incorporating FDA into the ensemble, we ensured methodological diversity beyond purely parametric (e.g., GLM) or additive (e.g., GAM) approaches. FDA’s flexible, spline-based regression architecture complements more constrained methods in the ensemble, reducing structural redundancy and enhancing overall explanatory power. Although it shares the general objective of classification with other methods, its foundational reliance on optimal scoring and non-linear basis expansions ensures a structurally distinct contribution to the ensemble forecast models.

GLM: Generalized Linear Model (14) extends classical linear regression to accommodate response variables that follow non-Gaussian distributions. Here, we applied GLMs with a binomial error distribution and a logit link function (native R *glm* function; version 4.3; [15]), effectively implementing logistic regression. This setup, widely used for presence-absence data, estimates the log-odds of species presence as a linear function of environmental predictors. GLMs comprise three components: the random component (distribution of Y), the systematic component (linear predictor), and the link function. Despite their simplicity, GLMs provide a robust, interpretable baseline and were used here to represent the parametric end of the modeling spectrum. They are advantageous in ecological modeling due to their interpretability and their ability to handle different types of response variables. GLMs allow for the assessment of the relationship between species occurrence and environmental factors, but their assumption of linearity may limit performance in the presence of complex relationships unless interaction or polynomial terms are included. In our ensemble, GLMs contribute a parametric perspective that complements non-parametric methods such as Random Forest and MaxEnt, enhancing robustness by incorporating varied assumptions and strengths.

GAM: Generalized Additive Model (16) is a semi-parametric generalization of GLMs that uses smooth functions (splines) to model nonlinear relationships between predictors and the response variable. The linear predictor is replaced by a sum of smooth functions, each estimated independently, allowing for flexible, non-linear, and non-monotonic species–environment relationships. GAMs were fitted with a binomial distribution and logit link using the *mgcv* package (version 1.8-42; [17]) in R (15). Smoothing parameters were estimated automatically via generalized cross-validation. In our study, GAMs were implemented using the mgcv package, which provides efficient algorithms for fitting GAMs with automatic smoothing. We employed a binomial error distribution with a logit link function to model species presence-absence data. Incorporating GAMs into the ensemble captures ecological complexity that may not be adequately modeled by purely parametric methods such as GLMs. Their inclusion enhances the ensemble's ability to generalize across different species and environments, improving both robustness and predictive performance.

RF: Random Forest (18) (via *randomForest* package version 4.7-1.1; [19]) is an ensemble learning method that constructs multiple decision trees using bootstrapped datasets and random feature subsets. Each tree provides a classification vote, and the final output is the majority vote (for classification) or the average (for regression). RF is robust to overfitting and captures complex interactions between variables. Its strengths include handling both continuous and categorical data, managing high-dimensional datasets, and coping with missing values. In the context of species distribution modeling (SDM), RF is particularly advantageous for modeling interactions and non-linearities. It also provides variable importance measures that aid ecological interpretation. In our study, RF was implemented with default parameters optimized for SDM tasks and trained on presence-absence data. The inclusion of RF enhances methodological diversity by offering a non-parametric, tree-based model that complements GLM, GAM, and MaxEnt.

MAXENT: Maximum Entropy Modeling of Species Niches (20, 21) is a presence-only algorithm that estimates a probability distribution of maximum entropy, constrained by the empirical average of environmental variables at observed locations. It uses multiple feature classes (e.g., linear, quadratic, hinge, product) and applies regularization to prevent overfitting. The model contrasts presence locations with background data to define suitable habitat. MaxEnt is particularly suited to species with limited occurrence records. In our study, MaxEnt was implemented using the standalone Java application (version 3.4.1, [22]), configured to generate response curves, perform jackknife tests, and produce logistic habitat suitability maps. Its inclusion enhances the ensemble by adding a robust presence-only method with broad ecological applicability.

MAXNET: Advanced Maximum Entropy Modeling (version 0.1.4) (23) is an R-native implementation of the MaxEnt algorithm, using the *glmnet* package (version 4.1-8; [24]) for elastic-net regularization. It supports the same feature classes as MaxEnt but integrates directly into the R environment, improving flexibility and reproducibility. In our study, Maxnet was applied using the maxnet package with environmental predictors and species occurrence data. It generated response curves, variable importance metrics, and habitat suitability maps. Including both MAXENT and MAXNET ensures methodological complementarity, as their differing implementations - Java-based versus R-native, with distinct regularization and optimization strategies - capture unique aspects of species–environment relationships and reduce algorithm-specific bias.

XGBOOST: Extreme Gradient Boosting (package version 1.7.6.1; [25]) is a gradient boosting algorithm that sequentially builds decision trees, with each tree correcting residuals from previous iterations. It uses first- and second-order derivatives of the loss function for optimization, improving computational efficiency and predictive performance. XGBoost applies L1 (Lasso) and L2 (Ridge) regularization to avoid overfitting and includes sparsity-aware techniques for handling missing data. In SDM, XGBoost is effective for modeling complex, non-linear species–environment relationships, particularly in high-dimensional and collinear datasets. It complements RF by introducing a boosting-based learning paradigm. Its inclusion improves the ensemble's ability to model intricate ecological patterns.

MARS: Multivariate Adaptive Regression Splines (26) (*mars* R package version 5.3.3, [27]) is a non-parametric regression method that models relationships using piecewise linear basis functions (hinges). It builds models in two stages: forward selection (adding basis function pairs that improve fit) and backward pruning (removing less informative terms using the Generalized Cross-Validation criterion). MARS can detect and model variable interactions automatically. This capability is valuable in SDM where environmental interactions shape species distributions. In our study, MARS was implemented using the earth package in R to model presence-absence data based on environmental predictors. Including MARS added flexibility and interpretability to the ensemble, particularly for non-linear and interactive effects that linear methods may miss.

Together, the inclusion of these eight algorithms ensures broad structural diversity and helps mitigate the risks of overfitting or model-specific artifacts. This multi-model strategy strengthens the ensemble’s generalizability across scenarios, taxa, and regions. The climate suitability modeling framework integrates insights and recommendations from multiple studies and adheres to best modeling practices (28-37). It employs the most accurate and up-to-date climate scenario campaign currently available for scientific studies: WCRP Coupled Model Intercomparison Project Phase 6 (CMIP6) (IPCC, 2023; 38) with high spatial resolution of 2.5 arc minutes (or cells with around 21.5 km² at the equator) at global coverage, excluding only massive water bodies and latitudes below -59.25° degrees. Baseline mathematical models were projected onto variables representing future climates across bi-decadal time horizons (2021-2040, 2041-2060, 2061-2080, and 2081-2100), all projected on the CMIP6 Shared Socioeconomic Pathway (SSP) 5-8.5 scenario (39). For this purpose, we selected three sets of General Circulation Models (GCMs) based on citation frequency in Scopus, as detailed in the following section.

*Climatic Variables*

For the baseline climate suitability scenario, 19 near-current bioclimatic variables (representing climate conditions from 1970 to 2000) were acquired from WorldClim (v2.1; https://www.worldclim.org; [7]). The initial list (before filtering) of baseline climate variables includes the following: BIO1: Annual Mean Temperature; BIO2: Mean Diurnal Range; BIO3: Isothermality; BIO4: Temperature Seasonality; BIO5: Max Temperature of Warmest Month; BIO6: Min Temperature of Coldest Month; BIO7: Temperature Annual Range; BIO8: Mean Temperature of Wettest Quarter; BIO9: Mean Temperature of Driest Quarter; BIO10: Mean Temperature of Warmest Quarter; BIO11: Mean Temperature of Coldest Quarter; BIO12: Annual Precipitation; BIO13: Precipitation of Wettest Month; BIO14: Precipitation of Driest Month; BIO15: Precipitation Seasonality (coefficient of variation); BIO16: Precipitation of Wettest Quarter; BIO17: Precipitation of Driest Quarter; BIO18: Precipitation of Warmest Quarter; BIO19: Precipitation of Coldest Quarter.

It is worth noting that several environmental and biological factors (e.g., land cover, vegetation types, biotic and abiotic barriers, environmental connectivity, physiological thresholds, availability of food, shelter/nest and reproductive resources) can influence the geographical suitability for the species and its invasive potential in new non-endemic areas. However, for this assessment, we did not find other environmental factors digitized on a global scale at high resolution that are known to influence *An. stephensi* specimens under baseline conditions and projected for future scenarios. To avoid modeling inaccuracies from the use of uncalibrated and non-significant variables that could lead to spurious relationships, we do not include any additional factors that might reduce the explanatory power of bioclimatic variables in expressing the effects of climate change on habitat suitability. Therefore, our results exclusively represent the species' climatic affinities and potential expansion limits under multiple specific scenarios.

Projections from GCMs generated for the same SSP5-8.5 often diverge among different climate modeling institutions. Determining which institution's estimates are more accurate remains unfeasible, as the real future climate is estimable but inherently uncertain. To ensure a thorough and robust selection, we adopted the criterion of utilizing GCMs most well-tested and frequently cited in the scientific literature. The analysis was performed using the R programming language, with the script and selection database provided in **Supplementary Data - Dataset S2**.

Firstly, we compiled the standard names of all GCMs available on the WorldClim portal ("ACCESS-CM2", "BCC-CSM2-MR", "CMCC-ESM2", "EC-Earth3-Veg", "FIO-ESM-2-0, "GFDL-ESM4", "GISS-E2-1-G", "HadGEM3-GC31-LL", "INM-CM5-0", "IPSL-CM6A-LR", "MIROC6", "MPI-ESM1-2-HR", "MRI-ESM2-0", "UKESM1-0-LL). This portal provides calibrated GCMs projected for different future periods (2021–2100), for specific SSPs, and provided at multiple resolutions. Calibrated GCMs are defined by geographic grids that perfectly align across variable sets for different periods and scenarios, maintaining identical extent and resolution, thus improving model comparability. Moreover, the geographic expression of climate change projected in future scenarios uses the same baseline (variables projected for 1970–2000) as the initial reference point. Therefore, variations in each variable due to estimated changes are added to or subtracted from the baseline variables. These calibrated variables enable an appropriate comparison between GCM projections, which are spatially expressed in the modeling outputs, as observed when comparing Ensemble Forecast Models (EFM) generated by GCMs from different institutions. We opted to acquire the variables from WorldClim portal because this source is widely recognized as providing robust and well-tested climatic layers and ranks among the most frequently cited resources in Scopus-indexed literature (www.scopus.com).

Subsequently, using sequential query searches in an R script with the *rscopus* package (40), all articles citing each GCM in the title, keywords, or abstract were retrieved from the scientific literature spanning 2018 to 2023. The total number of articles citing each GCM was then quantified. **Figure S5** illustrates the annual citation counts for each GCM, as well as the cumulative totals over the entire period.


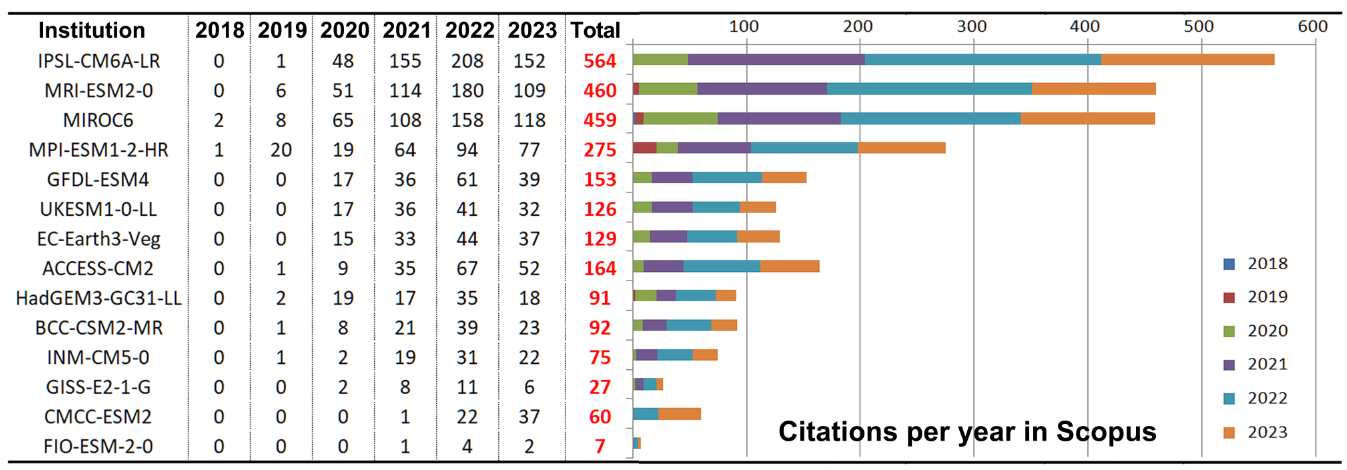


**Figure S5.** Most frequently cited GCMs from 2018 to 2023 from the Scopus Bibliographic Database (www.scopus.com).

Using this procedure, three GCMs were identified as the most frequently cited, standing out from the others. These models were developed by the following institutions: Institut Pierre-Simon Laplace (IPSL-CM6A-LR, cited 564 times; [41]), the Meteorological Research Institute (MRI-ESM2-0, cited 460 times; [42]), and the Model for Interdisciplinary Research on Climate (MIROC6, cited 469 times; [43]).

*Anopheles stephensi presences and pseudo-absence data*

We surveyed and compiled georeferenced presence records of *Anopheles stephensi*, both native and invasive, from virtual databases: (1) Malaria Atlas Project (44) and (2) GBIF (https://doi.org/10.15468/DL.VHGR3P); as well as from scientific literature, including Sinka *et al*. (2020; [45]), Tadesse *et al*. (2021 [46]), and Balkew *et al*. (2020 [47]). The dataset was refined through a three-step filtering procedure (**Figs. S6-A and S6-B**):

A) Exclusion of inaccurate or improbable records, such as those located over water bodies or in extreme arid desert environments devoid of human presence (validated visually via Google Earth), as well as records with poorly entered coordinates or imprecise georeferencing patterns.

B) Removal of duplicate records occurring within the same grid cells of environmental variables, as such redundancies do not enhance predictive modeling.

C) Exclusion of records from forested areas lacking visible signs of human habitation (confirmed visually via Google Earth).

This filtering process was designed to focus on occurrences with urban-affinity variants of *An. stephensi,* which are of primary interest to this analysis due to their role as vectors of urban malaria. Wild variants records, whose effect on malaria transmission is confined to natural habitats, were excluded as they are not relevant to the study and could weaken the predictions and inferences from the results. Our research aims to assess the human health risks posed by increased urban malaria transmission under climate change scenarios. Mosquito vectors with an affinity for urban environments represent the highest risk for human populations and are the focal point of this investigation.


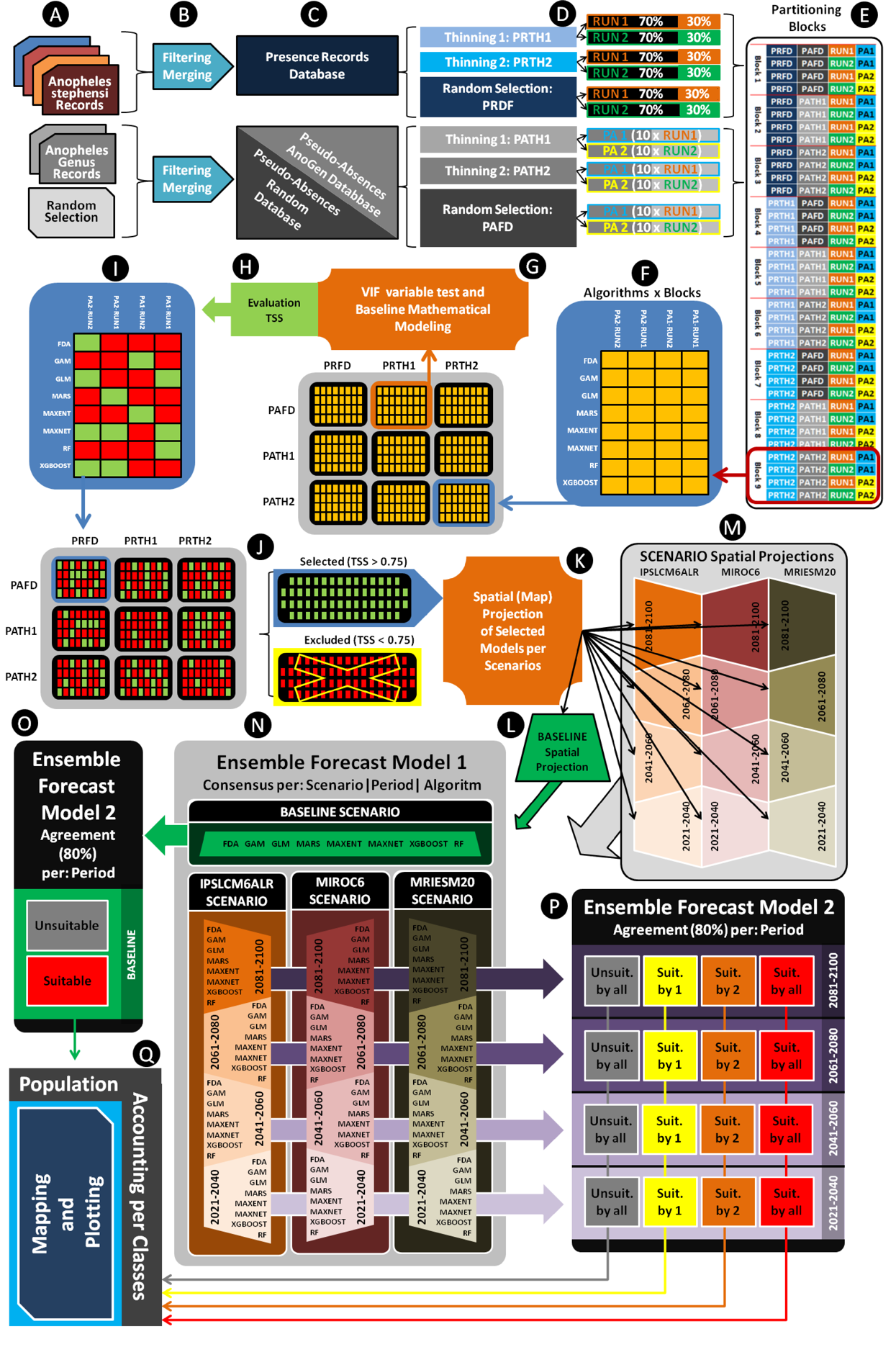


**Figure S6.** Flowchart of the modeling framework protocol.

Modeling algorithms requires geospatial data on species' empirical presences to identify environmental affinities within suitable ranges. Additionally, the algorithms rely on georeferenced data from environmentally unsuitable locations to delineate areas of non-suitability and to assess predictive performance (sensitivity and specificity rates). However, obtaining true absence data is inherently challenging, as it requires long-term continuous monitoring to confirm a species' absence at a given site. In this study, true absence data for *An*. *stephensi* was unavailable.

To address this demand, we adopted a plausible approach to estimate potential absence locations based on areas where other *Anopheles* species have been recorded, but *An. stephensi* has not. These areas serve as potential pseudo-absence sites for *An. stephensi*, as mosquito sampling/collection methods targeting *Anopheles* species would likely capture *An. stephensi* if present in the sampling site. The absence of *An. stephensi* in systematic field collections suggests its potential absence at those locations, although it may occasionally result from a lack of interest in reporting this species - a scenario deemed improbable in most cases. While locations with recorded presences of other *Anopheles* species but not *An. stephensi* cannot be definitively classified as true absences, they were utilized as pseudo-absences in the modeling process, contributing to the analysis with less weight than true absences.

Given that pseudo-absences carry less weight than true absences, a larger number is necessary to balance the high weight assigned to presence records modeling contribution. Following established recommendations (34, 21,48,49) and accounting for the global scale and high resolution of our approach, we adopted a strategy of using ten times more pseudo-absences than presences for each modeling round (49).

The pseudo-absences derived from *Anopheles* genus presence records, excluding *An. stephensi* (referred as Pseudo-Absences AnoGen Database; **Fig. S6-C**), may not be enough to meet the required number for multiple modeling rounds without replacement. To achieve the necessary quantity (approximately ten times the number of presences per round), additional pseudo-absences were generated randomly across the study area.

This process involved randomly selecting geographic coordinates across the climatic layer extent, ensuring a minimum distance of 20 km between points to avoid redundancy within the same climate grid cells, and maintaining at least 100 km from both *An. stephensi* presence records and PA-AnoGen locations. This distance far exceeds the maximum reported flight range for *Anopheles* species (50-52). These additional pseudo-absences are referred to as Pseudo-Absences Random Database (**Fig. S6-C**).

For each modeling round, we selected half of the required pseudo-absences from PA-AnoGen and the other half from PA-Random. This balanced approach ensured comprehensive global coverage, as PA-AnoGen records were predominantly concentrated in tropical zones, while PA-Random provided complementary worldwide spatial covering.

*Multi-blocks Partitioning Criteria*

To ensure accurate results that effectively inform medium- and long-term policy planning, the forecasts must precisely detect climate suitability in areas near native and invasive species presences as well as their surrounding climatically suitable regions. Importantly, these forecasts must robustly generalize and extrapolate predictions to distant regions lacking empirical presence data. Identifying all climatically suitable zones for this malaria vector, even in areas where the species has not yet been detected, is critical for enabling proactive and preventive measures in vector monitoring and control, thereby mitigating the risk or intensity of urban malaria transmission.

Consistent selection and partitioning criterion are crucial for enhancing the predictive performance of suitability models (53-55). To achieve this, input datasets were carefully randomized during each modeling round to minimize informational redundancy, reduce data clustering, and avoid prevalence effects. Two distinct partitioning approaches were employed to maximize input data variability and reduce geospatial clustering.

A) Thinning Partitioning Method: Following the Thinning method (56, 57) implemented using the *spThin* R package (58), the presence and pseudo-absence datasets were divided into different thinned subsets (Presences Thinned: PRTH1 and PRTH2; Pseudo-absences Thinned: PATH1, PATH2; **Fig. S6-D**). This process minimized risks of model overfitting and enhanced the generalization capacity of the EFMs by integrating predictions from multiple algorithms and modeling rounds using diverse datasets. Additionally, the complete presence and pseudo-absence datasets (Presence Full-Database - PRFD; Pseudo-absences Full-Database - PAFD; **Fig. S6-D**) were retained as sources for further partitioning.

B) *Biomod2* Embedded Random Partitioning: Utilizing the *Biomod2* package (version 4.2.2; [11]), the datasets underwent random partitioning tailored to the specific requirements of each modeling round. For presence data, 70% of records were allocated for model calibration, while 30% were used to test predictive quality based on the True Skill Statistic (TSS) (59). For pseudo-absences, the datasets were partitioned into subsets with ten times the number of presences, a strategy shown to optimize model performance (49). Before partitioning by *Biomod2*, the R sample function was used to pre-select a subset of pseudo-absences containing 20 times the number of presences, ensuring an approximate 50:50 balance between PA-Random and PA-AnoGen. This process results in three partitioned databases for presences in two random subsets each, and three partitioned databases for pseudo-absences in two random subsets each, totaling 12 partitioned databases, with six each for presences and pseudo-absences (**Fig. S6-D**).

To maximize input data variability, datasets were systematically combined into nine unique blocks containing 32 models, derived from the combination of input data subsets with algorithms (**Fig. S6-E**). Within each block, four models were generated per algorithm, resulting in a total of 36 models per algorithm across the nine blocks (**Fig. S6-F**).

*Framework Protocol*

The modeling process to generate the baseline scenario for *An. stephensi* climatic suitability, along with projections under future climate scenarios, was performed using the robust and widely validated *Biomod2* R package (version 4.2.2; [11]). *Biomod2* supports the use of multiple algorithms, each based on distinct mathematical principles, and provides tools for evaluating predictive quality, data partitioning, visualization, and other functionalities. The package also includes functions for generating EFMs by selecting and combining high-performing individual models (following quality assessments and the exclusion of weaker models), thereby improving overall predictive accuracy.

To streamline the description of framework specifics steps in this section, we have provided a flowchart in **Fig. S6** that synthesizes and visually outlines the sequential steps in the modeling framework, as described below:

(A) Compile data for all species within the *Anopheles* genus, including records for *An. stephensi*. Data were divided into two subsets: (1) occurrences of all *Anopheles* species except *An. stephensi*, and (2) records specific to *An. stephensi*. The first subset served as potential punctual absences (pseudo-absences) for *An. stephensi*, as these locations represented sites where *Anopheles* species were detected (typically using similar sampling/collection methods) but no *An. stephensi* specimens were found. The second subset, containing empirical occurrence records of *An. stephensi*, was used as the presence dataset (**Supplementary Data SI3 - Dataset S3**).

(B) The *An. stephensi* presence records (PR) dataset was filtered to remove duplicate entries with identical or closely overlapping coordinates, as well as dubious or improbable records, while accounting for other parameters. In addition to presence records, pseudo-absences (PA) were generated from records of other *Anopheles* species (AnoGen PA) and supplemented with randomly selected points across a global extent (Random PA) to meet the required quantity for each pseudo-absence subset (**Supplementary Data SI3 - Dataset S4**).

(C) Each presence record was assigned a unique ID and categorized for the initial partitioning process. Pseudo-absence data were classified as either those derived from the random selection process (PA Random Database) or those based on occurrence records of other *Anopheles* species (PA AnoGen Database).

(D) The R package *spThin* (58) was applied to both databases, producing thinned partitions for presence records (PRTH1 and PRTH2) and pseudo-absence records (PATH1 and PATH2) to minimize clustering effects within each subset. Additionally, the full presence (PRDF) and pseudo-absence (PAFD) datasets were retained for subsequent use in the random partitioning procedure using the embedded function in *Biomod2* package (version 4.2.2; [11]).

(E) Using the *Biomod2*, parameters were set to automatically and randomly partition each presence subset into two groups for each round: 70% of records were allocated to model generation and calibration via *Biomod2*’s embedded algorithms, while 30% were reserved to test model predictive quality using the TSS (59). Similarly, pseudo-absence records were randomly selected, totaling ten times the number of presence records per modeling round, from thinned and random subsets (PA1 and PA2), and applied in each respective modeling round. This procedure, which combines multiple partitioned datasets, creates 36 unique configurations across 9 distinct blocks. This dataset multi-partitioning approach ensures significant variability and randomness in presence and pseudo-absence subsets for the multi-modeling approach, thereby optimizing model generalization and enhancing predictive accuracy (Models outputs per block available in **Supplementary Data SI3-**  **Dataset S5**).

(F) This step is illustrated by an example fragment from one of the 9 generated blocks (PRTH2xPATH2) dedicated to the multi-algorithm modeling process conducted using 8 different algorithms (listed as row names). For each block, 32 models are generated, with 4 models produced per algorithm.

(G) Each partitioned set of presence data (RUN1 and RUN2), together with the 19 climate variables, underwent a Variance Inflation Factor (VIF) test to identify and exclude variables with collinearity issues. This step is essential to mitigate the adverse effects of collinearity among highly correlated spatial variables in regression-based modeling algorithms. This test evaluates the R-squared value of a regression for each variable against all others in a stepwise procedure. Variables exceeding the predetermined VIF threshold were removed, and the VIF values were recalculated iteratively for the remaining variables until achieving a set with minimal risk of multicollinearity. For this analysis, we utilized the *vifcor* function from the *usdm* R package (60), retaining only variables with a VIF correlation threshold below 0.95 for the modeling procedure. To enhance accuracy, our VIF test did not rely on randomly sampled variables from the global extent but instead incorporated the geographical positions of species occurrences for each modeling round. The VIF test was conducted using the subset of presences selected in each iteration, combined with a random sample of 10,000 values from each environmental layer. Sampling was constrained to a 100 km radius (buffer) around the presence records for each subset in the modeling round. This approach ensures that the collinearity measure is geospatially specific and dependent on the species distribution, thereby providing a more robust test for addressing collinearity effects. The VIF values and the final selected variables for each block are documented in the folder corresponding to each modeling block (VIF test outputs available in **Supplementary Data SI3 - Dataset S6**).

(H) All 288 models generated for the baseline scenario (4 PAxRUN x 9 blocks x 8 algorithms = 288) are subsequently evaluated using the TSS predictive quality index (59).

(I) The example evaluation block (PRTH1xPAFD, highlighted in orange square in step (G) demonstrates the model selection process following TSS evaluation. Green squares inside this block (I) represent models with high predictive quality (TSS > 0.75), while red squares indicate models with insufficient predictive quality. Although a perfect model achieves a TSS of 1, this is rarely attained in practice, and models with TSS < 0.5 are considered to have low predictive capacity. To ensure robust results, only models with TSS values above 0.75 are selected for subsequent combination into EFMs.

(J) This frame shows the total evaluation of the 288 models generated across the nine blocks, where only a subset (green squares, indicating models with TSS > 0.75) is retained for the subsequent stages of analysis. The remaining models (red squares, representing models with TSS < 0.75) are excluded from further consideration.

(K) The models that passed the TSS quality test are spatially projected using a specific *Biomod2* function, which translates the numerical outputs of the mathematical models into geographic representations. At this stage, the models are visualized spatially by plotting the resulting raster data in a mapped planar format.

(L) The planar projection process (from mathematical models) is first conducted for the baseline scenario, utilizing the set of variables selected through the VIF test. These variables are applied both in the mathematical modeling by algorithms and in the spatial projection of the model results.

(M) The projections for future periods are generated using the baseline mathematical models and the new values of the same (meaning) variables selected after the VIF test. These projections spatially represent climates in future periods (2021-2040, 2041-2060, 2061-2080, 2081-2100) as estimated and projected by using GCMs from each institution: IPSL-CM6ALR, MIROC-6, and MRI-ESM20. As with the baseline scenario, only models that met the minimum TSS threshold (TSS > 0.75) were projected under selected GCMs variables for future scenarios.

(N) EFMs were generated using the Committee Averaging Score within the *Biomod2* package (11, 12, 61). This method transforms probabilistic predictions from individual models into binary predictions using specific cut-off thresholds (35). In this implementation, the EFM's geospatial output does not depict climatic suitability levels directly but rather the level of agreement among robust prediction rounds, yielding an integrated, mapped outcome. In this first batch (EFM1 in **Fig. S6-N**) one EFM was generated for each algorithm in every grouping by scenarios and periods (e.g., MIROC6 > 2041-2060 > MAXENT). Following the generation of EFMs, a refinement threshold process was also applied: only areas with at least 80% agreement in suitability predictions across algorithms and rounds were classified as suitable, while the remaining 20% with lower concordance were deemed unsuitable. This threshold, determined through sensitivity analysis (details in the next topic: *Sensitivity Analysis to Determine Cutoff Threshold Refinement*), effectively trims marginal prediction edges (from EFM batch 1 results) where intrinsic error is likely higher, thereby improving the accuracy of the final EFM outcomes (EFM batch 2).

(O and P) At those stages, algorithm-specific EFMs (EFM1 in Fig S4-N) are then aggregated by periods (2021-2040, 2041-2060, 2061-2080, 2081-2100), producing the second batch of ensemble models (EFM2): one for the baseline (**Fig. S64-O**) and five for each future period (**Fig. S6-P**). It is important to note that the EFM generated for the baseline scenario was constructed using a single set of selected near-current climate variables (1970–2000), chosen after the VIF test. Consequently, the baseline EFM2 (**Fig. S6-O**) contains only two binary classes: suitable or unsuitable. In contrast, the EFMs generated for future scenarios (**Fig. S6-P**) include five classes derived from cumulative suitability "votes" among EFMs, based on the level of agreement in suitability predictions (overlaps) from the EFMs produced by the three GCM sets, one from each institution:

**Consensus Suitability Forecast (CSF)**: Represents areas consistently predicted as suitable by all EFMs for each future period. This zone reflects full spatial overlap and complete agreement on indicating climatic suitability, signifying three unanimous votes of suitability from EFMs based on the GCMs sets of the three institutions (red squares, Fig. S4-P). The CSF covers a more geospatially-restricted extent compared to other classes. The CSF, by focusing exclusively on suitable regions equally predicted by all EFMs (using variables from all GCMs-institutions), defines a more spatially restricted area but with a higher confidence level (zones of maximum probability/quality in suitability prediction), not including marginal areas with conflicting predictions among EFMs, where intrinsic error may be higher. Prioritizing this extent is thus optimal for maximizing the chances of detecting and observing *An. stephensi* in the field. In this way, the CSF is particularly useful for prioritizing emergency control and impact mitigation actions, especially when resources allocated to vector control are limited. Its narrower spatial extent (compared to GSF) enables a concentrated focus on areas with the highest statistical likelihood of higher quality of suitable climate conditions for the vector.

**Consensus Unsuitability Forecast (CUF)**: Represents areas consistently predicted as unsuitable by all EFMs, integrating all GCMs from the three institutions. This indicates full spatial overlap and agreement on climatic unsuitability (gray squares, **Fig. S6-P**). This zone is crucial for spatially indicating the climatic conditions that limit the species' distribution expansion, and possibly, where the risk of urban malaria transmitted by *An. stephensi* is lower.

**Single Agreement Forecast (SAF)**: Represents areas predicted as suitable by EFMs from only one institution (one EFM vote in suitability), regardless of which institution (yellow squares, **Fig. S6-P**). This SAF class, although constructed, calculated and mapped, was not analyzed individually but as part of the General Suitability Forecast.

**Double Agreement Forecast (DAF)**: Represents areas predicted as suitable by EFMs from any two institutions (two EFM votes in suitability), regardless of which institutions (orange squares, **Fig. S6-P**). In the same manner as the SAF, the DAF class, although constructed, calculated, and mapped, was not analyzed individually but was analyzed incorporated into the General Suitability Forecast.

**General Suitability Forecast (GSF):** Represents areas identified as suitable by at least one EFM using GCM from any institution (receiving a minimum of one EFM vote for suitability, up to a maximum of three votes). The GSF encompasses the combined spatial extents of SAF, DAF, and CSF, making it the most geospatially comprehensive zone of climatic suitability for *An. stephensi* (combined yellow, orange, and red squares; not isolated depicted in **Fig. S6**). The GSF may be suited for prioritizing areas for large-scale, long-term monitoring, planning preventive measures, and conducting awareness and prevention campaigns, as it incorporates the combined 'strengths' of explanatory variables from all institutions to generate the EFMs. Its broader scope, which equally weights zones of prediction convergence and divergence among GCMs, reduces the likelihood of omission errors in selecting priority areas for intervention. While we cannot determine which institution's predictions most robustly capture future climate conditions and thus contribute the most accurate suitability models, including suitability predictions from all GCMs substantially increases the likelihood of covering the zones most predicted as suitable for one or more “institutions” (EFMs from GCMs).

(Q) EFM classes were cross-referenced with population data from NASA-SEDAC (1) that provide spatial estimates of human populations within each grid cell, globally. Using zonal statistics techniques into Geographical Information Systems, we calculated the population within the overlapping areas of each EFM2 class. This population estimate was then adjusted based on the United Nations World Population Prospects (2), and the coverage area of each class (from NASA-SEDAC) was recalculated. To conduct numerical analyses based on official demographic indices by country (country area, population size, and density), we used data provided by the World Population Prospects 2024 from the United Nations Department of Economic and Social Affairs - Population Division (2). Malaria cases and mortality rate data by country were sourced from the World Malaria Report (62).

*Sensitivity Analysis to Determine Cutoff Threshold*

This procedure was implemented to refine the results of the initial batch of models comprising the EFM (Araujo and New, 2007; **Fig. S6-N**) by eliminating marginal zones divergently predicted as suitable by some of models (from different algorithms) that demonstrated lower consistency across the majority of modeling iterations. These marginal zones are more prone to statistical artifacts and inaccuracies. By trimming them, the procedure minimizes potential errors without significantly compromising sensitivity (true positive rate) or affecting specificity (true negative rate) (59, 63, 64). Additionally, this method reduces zones of potential over-extrapolation, often contributed by model rounds with TSS values above but near the minimum acceptable threshold of predictive quality (TSS > 0.75; [59]). While these models meet minimal quality standards, their predictions are slightly less reliable compared to others in the overall ensemble context.

The rationale for this sensitivity analysis is grounded in tests conducted by Liu *et al*. (2013 [63]) and builds upon the results and recommendations of prior studies (65-67). The process adheres to the metric assumptions of the TSS index (59). It is important to emphasize that this cutoff does not exclude entire models or large suitable area predictions from the EFM but targets marginal strips of predicted climatic suitability with very low agreement levels across modeling rounds from each algorithm. The objective was to reduce marginal areas predicted as climatically suitable while preserving the ability of the remaining areas to accurately indicate suitability, as measured by the hit rate of true positives (sensitivity).

To accomplish this, the baseline models that passed the TSS evaluation (TSS ≥ 0.75) were utilized (N = 112 out of 288 generated). These models were applied in binary mode (0 = unsuitable; 1 = suitable), with cutoff thresholds parameterized using the *Biomod2* `*CutOff.Optimised*` function (11) to achieve an optimal balance between specificity and sensitivity. This parameterization minimizes discrepancies between sensitivity and specificity for each modeling round (63, 67) and ensures a balanced geospatial representation by maximizing sensitivity (accurately identifying suitable areas where presences occur) and specificity (accurately identifying unsuitable areas where pseudo-absences occur). This approach mitigates the risks of overfitting and underfitting, enhancing both predictive generalization and accuracy.

The presence hit rate (PHR) was evaluated using the complete presence records dataset (N = 756). Binary models were spatially stacked to calculate predictive agreement by summing overlapping rasters, creating a raster akin to a geospatial histogram. This raster was intersected with the presence database to assess how many presences were covered by each predictive agreement class (**Table S1**, N. Presence Hits column). Additionally, the spatial extent of each class, including unsuitable cells, was calculated to estimate the coverage of each class (**Table S1**, Suitable Cells and Unsuitable Cells columns).

**The Figure S7** was created to observe and analyze the progressive variation of the variable Presence Hit Rate (PHR; values associated with the left Y-axis) and the respective Delta PHR (values associated with the right Y-axis) according to the downward shrinkage/decrease of the spatial extent of the climate suitability area represented by the variable Suitable Area Extent % (SAE; values associated with the left Y-axis) and the respective Delta SAE (values associated with the right Y-axis) with the simultaneous upward increase in the level of agreement among models (variable N. Model Agreement).

**
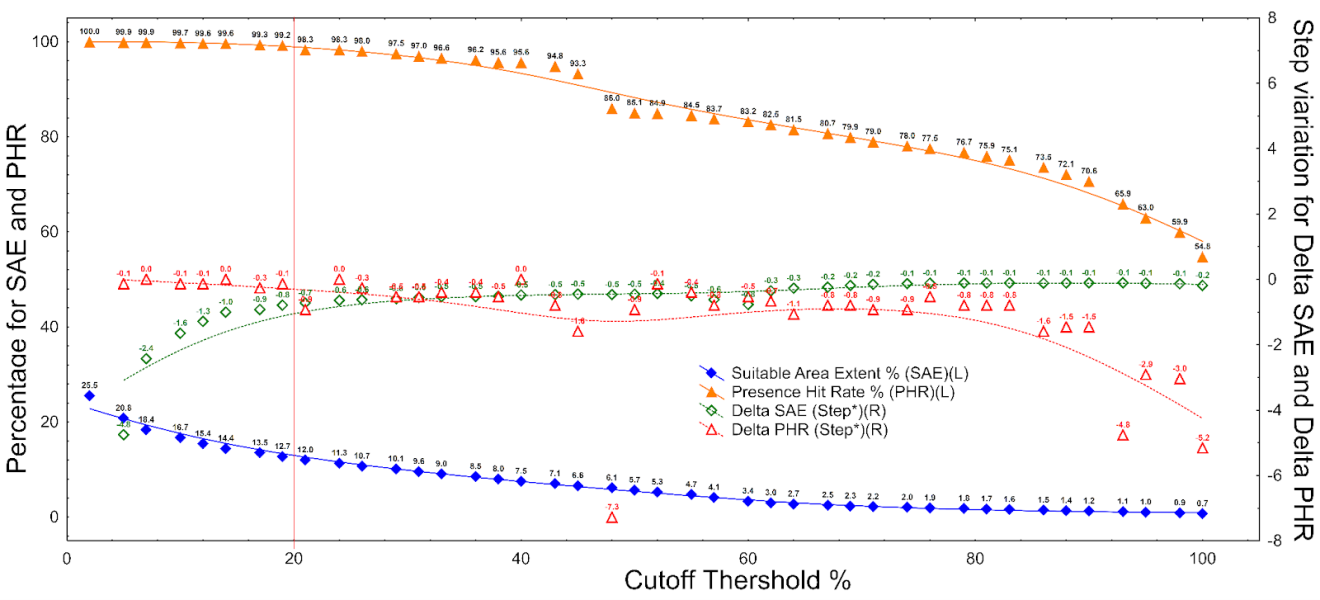
**

**Figure S7.** Behavior of variables in response to the progressive cutoff threshold (%) shown on the X-axis. The variables are as follows: Suitable Area Extent (% SAE) and Presence Hit Rate (% PHR), represented by lines associated with the left Y-axis. Delta SAE and Delta PHR, represented by lines associated with the right Y-axis. The plot highlights the progression of these variables as the cutoff threshold increases. At the 20% point on the X-axis, the red line marks the high-sensitivity threshold, where a sharp drop in Delta PHR is observed immediately after 20% (indicated by the red triangle). This abrupt change signals the optimal stopping point for the sensitivity analysis. It represents the balance between sufficient reduction of marginal areas and maintaining predictive accuracy, ensuring minimal impact on the predictive accuracy.

It was observed that the initial SAE represents 25.5% of the total spatial extent of bioclimatic variables (at worldwide coverage), and the stepwise progressive spatial shrinkage ceases when SAE reaches 12.7% (Suitable Area Extent in **Table S1**). At this point, we observe an abrupt decrease in the presence hit rate (column PHR- Presence Hit Rate %) from 99.21% (at line ID-Name: NMA8.P19) to 98.28% (at line ID-Name point: NMA9.P21), with a decrease of -0.93%. Note that in the Delta PHR sequence, the spatial reduction of SAE in the previous steps varied between 0 and -0.13%, with a maximum decrease of -0.26% (Delta PHR at point NMA7.P17). This relatively intense decrease of -0.93% represents a reduction of approximately 5.7% in SAE compared to the previous step, and a reduction of approximately 53% in SAE compared to the starting point SAE value (SAE at line NMA1.P2). Due to this significant response in PHR and the large area reduction of SAE, it was concluded that the process should cease at this point of ~20% of the Cutoff Threshold. Therefore, the area of climate suitability predicted by only 20% of the models (from different algorithms and rounds) that were least in agreement with the remaining 80% was reclassified as unsuitable for subsequent analyses.

**Table S1***.* Sensitivity Analysis Tabular Results: Summarizes multiple calculations derived from the overlap of selected models after TSS evaluation to assess the level of predictive agreement and the extent of each class based on grid cell counts. Key indicators are presented in the highlighted columns: N. Model Agreement: The number of binary models whose suitability predictions overlap within the same geographical space. Presence Hit Rate (% PHR): The proportion of species occurrences covered by all predictive classes, including those with lower agreement. This is calculated as the ratio of N Presences Hits (PH) to N Presences Misses. The maximum PHR is achieved when all selected models (post-TSS) are considered collectively. The table also shows the progressive shrinkage of suitable zones, achieved by excluding marginal areas with lower agreement in suitability predictions. This shrinkage results in a gradual reduction of the number of presence records intercepted within suitable zones, thereby decreasing the PHR. Suitable Area Extent % (SAE): Represents the proportion of grid cells predicted as suitable (Suitable Cells) relative to those predicted as unsuitable (Unsuitable Cells) within each agreement class. Cutoff Threshold (%): Indicates the percentage of models that predicted suitability within the respective marginal zone excluded at each step (SAE). In this analysis, the cutoff threshold was observed between 19% and 21%, defined as 20% for consistency. Delta Variables (Delta PH, Delta PHR, Delta SAE) represent the differences between consecutive steps, showing how much each variable (PH, PHR, and SAE) changes at each step. Delta PHR is particularly critical for detecting significant shifts in the Presence Hit Rate, aiding in the identification of the most appropriate cutoff threshold.


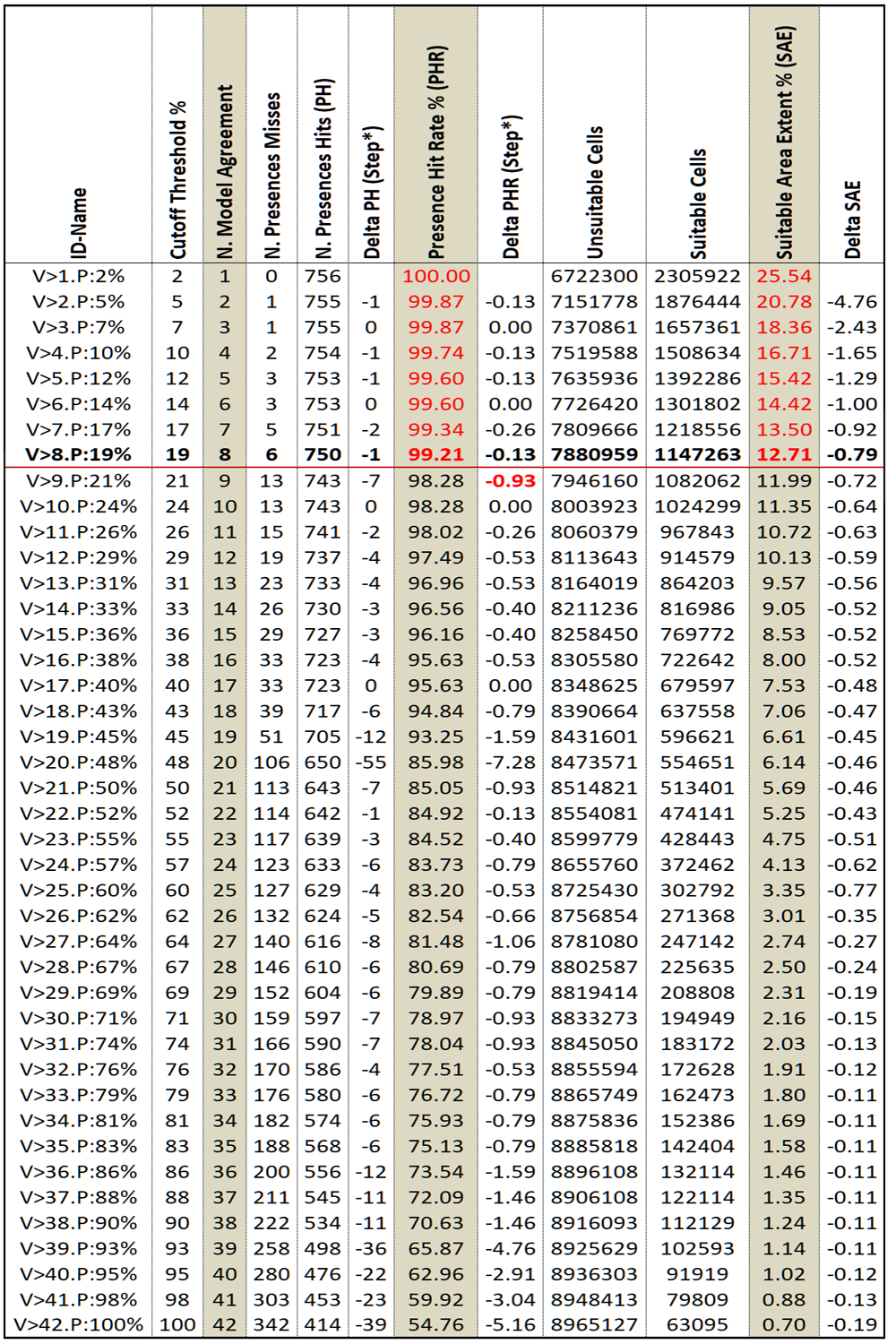


*Vulnerable Human Population Accounting*

To account for the number of humans covered by each climate suitability class for *An. stephensi* in multiple periods, we utilized geospatial projections provided by NASA-SEDAC (1) generated for the SSP5-8.5 pathway, which correspond to the pathway used in the GCMs for our models. The NASA-SEDAC demographic projection layers provide estimates for the decades from 2010 to 2100. CMIP6-GCMs are presented in two-decade periods, such as 2021-2040. Since the geographical expression of GCM variables represents the climatic outcome of the last year of each period, the corresponding demographic layers were selected for each final decade per climate period. For example, to account for the vulnerable human population in EFMs generated using GCMs for the period 2021-2040, we used the demographic layer for 2040. For the baseline scenario, representing the climate between 1970 and 2000, we used the 2010 demographic layer for calculations, as there were no estimates available for earlier periods in the NASA-SEDAC dataset.

For the calculation, we converted the pixels of each demographic/population layer (for each respective decade) from their original raster format (GeoTIFF) to a point shapefile using the *rasterToPoints* function of the *raster* package (version 3.5-11; [68]) in R (15,), retaining the population values assigned to each point. We then extracted the suitability class values of the EFMs (for each respective period) for the point shapefile using the *extract* function in R (68). The data were aggregated to obtain the values for the number of people in each climate suitability class. This procedure was performed separately for each scenario and each period.

To obtain a second estimate that could reveal possible population underestimation in relation to the most recent demographic projections, we adjusted the population values for each suitability class with the estimates provided by United Nations World Population Prospects (2) for comparison. We used the proportion of the global population covered by each suitability class to estimate absolute values. In other words, if a particular class (for a specific scenario and period based on NASA-SEDAC) covers 20% of the global population, we obtained the absolute value for this percentage from the UN projection (estimated for the same specific decade). Although this simplification is a limitation, it provides an adequate approximation to assess the difference between the projections of the two institutions (demographics data provider) and evaluate the level of risk that the expansion of *An. stephensi* poses to the global population.

*Overall Modeling Assessment*

The modeling framework generated 288 models in the baseline scenario, of which 112 models were selected for their high predictive quality (TSS >= 0.75), while the remaining 176 baseline models were excluded. Consequently, we projected 112 models for each future scenario, totaling 1,456 models (**Figs. S8-A to S8-E**).


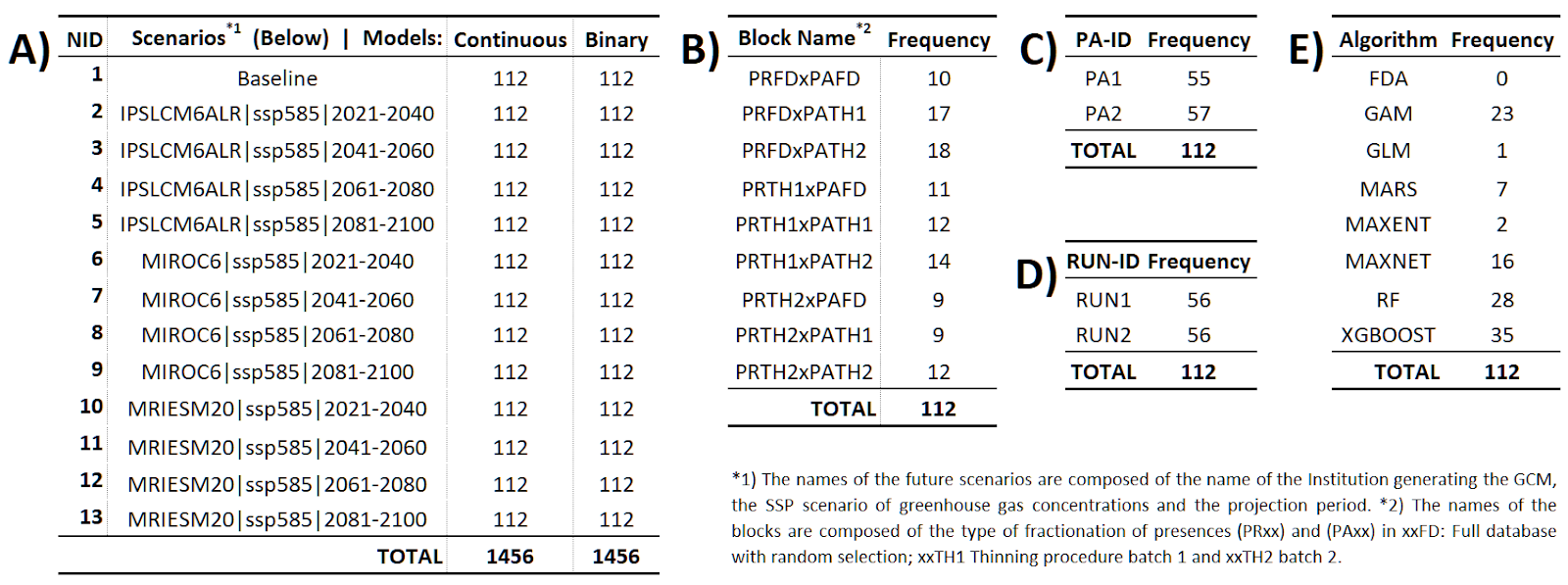


**Figure S8.** Accounting of multiple characteristics from selected models (TSS ≥ 0.75) and their respective scenarios, algorithms, blocks, and input data partitioning. (A) Accounting of selected scenarios (Institution | SSP pathway | Period) and models after TSS ≥ 0.75 in continuous mode and subsequent reclassification in binary mode. (B) Accounting for the number of selected models for each block. (C) Accounting for selected pseudo-absences subsets. (D) Accounting for partitioning rounds. (E) Accounting to detect the best-performing algorithms.

The results indicated the superiority of the Extreme Gradient Boosting algorithm (XGBOOST; **Fig. S8-E**), which provided models with high predictive quality, representing approximately 31% of the total. This was followed by Random Forest with 25% (RF) and Generalized Additive Models with around 20% (GAM). The worst-performing algorithm was Flexible Discriminant Analysis (FDA), with no models selected. Following FDA, the Generalized Linear Model (GLM; 0.9%) and Maximum Entropy Modeling (Maxent; 1.8%) algorithms performed poorly, contributing only 1 and 2 models, respectively (from 112 models at baseline). Interestingly, the new MAXNET algorithm (23), an open-source R implementation based on the original closed-source JAVA-based MAXENT algorithm by Phillips *et al*. (2006; [20]), outperformed its predecessor by generating approximately 15% of the selected models.

Among the approaches to partitioning presences and pseudo-absences, the combination of random presence selection from the full dataset (PRFD) with pseudo-absence selections using the Thinning technique (PATH2 and PATH1) contributed most to high-quality predictive models, accounting for approximately 16% and 15% of models, respectively (**Fig. S8-B**). In contrast, the poorest-performing combinations involved thinning on the second subset of presences (PRTH2) with the first subset of pseudo-absences thinning (PATH1) and with random selection of the total set of pseudo-absences (PAFD), each contributing only 8% of the total models.

This overview provides a general assessment of the modeling framework performance. Our primary aim is to generate robust models to demonstrate the expansion of the climatic suitability of *An. stephensi* across multiple scenarios, rather than to compare the predictive quality among algorithms, models, and input partitioning data subsets. Nonetheless, we provide the modeling inputs and outputs in the **Supplementary Data SI3 - Datasets S1, S4** and **S5** for further consultation.

*Variable Importance and Climatic Profile Analysis*

To identify and measure the main factors shaping the global distribution of *An. stephensi*, we performed a two-part analytical procedure under baseline climate conditions - since future climate scenarios are projected relative to these baseline ranges in CMIP6-SSP models: (A) quantification of the relative importance of environmental predictors in the modeling framework, and (B) extraction and analysis of the empirical ranges of these variables across areas predicted as suitable for the vector. The variables were interpreted within four biologically relevant domains for mosquito survival and malaria transmission: rainfall, temperature, relative humidity, and altitude.

Variable importance assessment was based on outputs from 112 selected ensemble models generated under the multi-algorithm framework of the *biomod2* package (v.4.2.2) in R (v.4.3.1) (11). Only model runs with a True Skill Statistic (TSS) ≥ 0.75 were retained to ensure high predictive accuracy.

For each run, *biomod2* computes variable importance scores (available in Dataset S5) by quantifying prediction degradation under randomized permutations (N:5) of each predictor. These importance scores were aggregated across all eligible models, and descriptive statistics (mean, standard deviation, median, and quartiles) were computed for each variable. Predictors were then classified into four categories - Maximum, High, Moderate, and Low importance - based on quartile thresholds (25th, 50th, 75th percentiles) of the overall distribution. All processing steps were scripted in R using the *dplyr* (version 1.1.0.; [69]), *pbapply* (version 1.6-0; [70]), and *ggplot2* (version 3.4.4; [71]) packages.

Climatic profile extraction was conducted post hoc, based on modeling results, to enhance the interpretability of predictor influences in suitability forecasts. Binary raster outputs from the ensemble baseline model were used to isolate areas classified as suitable (value = 1). For these grid cells (value = 1), we extracted intersecting values from all 19 bioclimatic variables and elevation (7). We also extracted baseline values for CHELSA-derived (8) humidity variables (minimum, mean, maximum, range). Relative humidity variables, although not included in the modeling due to the indisponibility of well-tested harmonized projections for future climate scenarios (SSP-CMIP6), were analyzed post hoc by extracting their baseline ranges in suitable areas to provide insights into the species’ ecological tolerance to atmospheric moisture.

Extraction was performed using the *raster* (version 3.5-11; [68]) and *sp* (version 1.6-0; [72]) packages in R. Each variable’s distribution across suitable areas was summarized using five-number statistics (min, Q1, median, Q3, max) and visualized via boxplot panels. This procedure allowed us to contextualize the predictors’ statistical importance with the environmental conditions where the species are and may establish in future, offering biologically grounded interpretation of habitat suitability under current climate conditions. All outputs and R scripted codes can be assessed in Dataset S8.

*Ecological and Evolutionary Caveats*

Our projections reflect climatic suitability based solely on bioclimatic variables and presence-based modeling. However, several ecological and evolutionary mechanisms not explicitly modeled may shape the realized distribution of *An. stephensi* These include: land use and land cover dynamics, vegetation structure, landscape fragmentation, environmental connectivity, availability of aquatic breeding sites, competition with local vector species, and natural or anthropogenic barriers to dispersal.

Moreover, *An. stephensi* exhibits a high degree of phenotypic plasticity, behavioral flexibility, and short generation time - all of which can accelerate adaptation to novel environments. Urban ecosystems, in particular, may facilitate genetic selection for traits enhancing thermotolerance, insecticide resistance, or desiccation resistance. Although our models are calibrated to empirical occurrence records, the species may evolve to exploit climatic conditions beyond its present physiological niche through rapid adaptation or niche shifts.

Thus, the projected suitability maps should be interpreted as conservative estimates, representing current climatic constraints. Real-world dynamics may deviate from modeled projections due to both ecological barriers and adaptive responses, especially under continued climate change and urban expansion.

**SI3) Supplementary Data**

All datasets can be downloaded from: A. Acosta, Future Global Distribution and Climatic Suitability of Anopheles stephensi. Zenodo. **https://doi.org/10.5281/zenodo.15558088**.

**Dataset S1.** Full database of demographics and modeling results.

**Dataset S2.** R Script and database for Selecting GCMs Based on Citation Frequency from Scopus.

**Dataset S3.** Presences Database (point shapefile) of Anopheles stephensi occurrences (LocType=PU: Peri-urban; LocType=U: Urban).

**Dataset S4.** Pseudo-Absences Database (point shapefile) of Anopheles species except Anopheles stephensi (Type=PA_Anogen) and randomized selected pseudo-absences (Type=PA_Random).

**Dataset S5.** Models outputs per blocks.

**Dataset S6.** VIF test outputs per blocks.

**Dataset S7.** R Language Scripted Codes of Analysis and Modeling.

**Dataset S8.** R Language Scripted Codes of Variables Importance and Ranges.

**SI4) Supplementary References**

1. Jones, B. & O'Neill, B. C. Global one-eighth degree population base year and projection grids based on the shared socioeconomic pathways, revision 01. NASA Socioeconomic Data and Applications Center (SEDAC) (2020).
2. United Nations, Department of Economic and Social Affairs, Population Division. World Population Prospects 2024: Summary of Results (UN DESA/POP/2024/TR/NO. 9) (2024). Available at:<https://population.un.org/wpp/publications/>
3. Carter, T. E. *et al.* The ecology of container-inhabiting mosquitoes in urban settings. *J. Med. Entomol.* 57, 887–899 (2020).
4. Balkew, M. *et al.* Detection of *Anopheles stephensi* in Ethiopia using molecular tools. *Acta Trop.* 188, 180–186 (2018).
5. Sinka, M. E. *et al.* A new malaria vector in Africa: Predicting the expansion of *Anopheles stephensi*. *Lancet Planet. Health* 4, e601–e609 (2020).
6. Tadesse, F. G. *et al.* The threat of *Anopheles stephensi* to the Horn of Africa – a new challenge for malaria elimination. *Lancet Infect. Dis.* 21, 1378–1379 (2021).
7. Fick, S. E. & Hijmans, R. J. WorldClim 2: New 1 km spatial resolution climate surfaces for global land areas. *Int. J. Climatol.* 37, 4302–4315 (2017).
8. Karger, D. N. *et al.* Climatologies at high resolution for the earth’s land surface areas. *Sci. Data* 4, 170122 (2017).
9. De Zulueta, J. Mosquito ecology and malaria transmission. *WHO Monogr. Ser.* No. 30 (1959).
10. Surendran, S. N. *et al.* Anthropogenic impact on urban malaria risk in Sri Lanka: the role of invasive *Anopheles stephensi*. *Parasit. Vectors* 15, 64 (2022).
11. Thuiller, W., Georges, D., Engler, R. & Breiner, F. biomod2: Ensemble platform for species distribution modeling. R package version 4.2.2 (2023). Available at: [https://CRAN.R-project.org/package=biomod2](https://cran.r-project.org/package=biomod2).
12. Thuiller, W., Lafourcade, B., Engler, R. & Araújo, M. B. BIOMOD – A platform for ensemble forecasting of species distributions. *Ecography* 32, 369–373 (2009).<https://doi.org/10.1111/j.1600-0587.2008.05742.x>
13. Hastie, T. & Tibshirani, R. mda: Mixture and Flexible Discriminant Analysis. R package version 0.5-5 (2024). Available at:<https://cran.r-project.org/package=mda>.
14. McCullagh, P. & Nelder, J. A. *Generalized Linear Models*. Springer US (1989).
15. R Core Team. R: A language and environment for statistical computing. Version 4.3.1. R Foundation for Statistical Computing, Vienna, Austria (2021). Available at: [https://www.R-project.org](https://www.r-project.org/).
16. Hastie, T. J. & Tibshirani, R. J. *Generalized Additive Models*. Chapman and Hall, New York (1990).
17. Wood, S. N. mgcv: Mixed GAM Computation Vehicle with Automatic Smoothness Estimation. R package version 1.8-42 (2023). Available at:<https://cran.r-project.org/package=mgcv>.
18. Breiman, L. Random forests. *Mach. Learn.* 45, 5–32 (2001).<https://doi.org/10.1023/A:1010933404324>.
19. Liaw, A. & Wiener, M. Classification and Regression by randomForest. *R News* 2, 18–22 (2002). R package version 4.7-1.1 (2024). Available at:<https://cran.r-project.org/package=randomForest>.
20. Phillips, S. J., Anderson, R. P. & Schapire, R. E. Maximum entropy modeling of species geographic distributions. *Ecol. Model.* 190, 231–259 (2006).<https://doi.org/10.1016/j.ecolmodel.2005.03.026>.
21. Phillips, S. J., Dudík, M. & Schapire, R. E. Sample selection bias and presence-only distribution models: Implications for background and pseudo-absence data. *Ecol. Appl.* 19, 181–197 (2009).<https://doi.org/10.1890/07-2153.1>.
22. Phillips, S. J. MaxEnt software for modeling species niches and distributions. Version 3.4.1 (2017). Available at:<https://biodiversityinformatics.amnh.org/open_source/maxent/>.
23. Phillips, S. J., Elith, J., Hastie, T. & Dudík, M. maxnet: Fitting ‘Maxent’ Species Distribution Models with R. R package version 0.1.4 (2022). Available at:<https://cran.r-project.org/package=maxnet>.
24. Friedman, J., Hastie, T. & Tibshirani, R. Regularization Paths for Generalized Linear Models via Coordinate Descent. *J. Stat. Softw.* 33, 1–22. R package version 4.1-8 (2023). Available at:<https://cran.r-project.org/package=glmnet>.
25. Chen, T. & Guestrin, C. XGBoost: A scalable tree boosting system. In *Proceedings of the 22nd ACM SIGKDD International Conference on Knowledge Discovery and Data Mining*, 785–794 (2016). R package version 1.7.6.1 (2024). Available at:<https://cran.r-project.org/package=xgboost>.
26. Friedman, J. H. Multivariate adaptive regression splines. *Ann. Stat.* 19, 1–67 (1991).<https://doi.org/10.1214/aos/1176347963>.
27. Milborrow, S. earth: Multivariate Adaptive Regression Splines. R package version 5.3.3 (2024). Available at:<https://cran.r-project.org/package=earth>.
28. Levine, R. S., Peterson, A. T. & Benedict, M. Q. Geographic and ecologic distributions of the *Anopheles gambiae* complex predicted using a genetic algorithm. *Am. J. Trop. Med. Hyg.* 70, 105–109 (2004).
29. Elith, J., Leathwick, J. R. & Hastie, T. Novel methods improve prediction of species’ distributions from occurrence data. *Ecography* 29, 129–151 (2006).
30. Ortega-Huerta, M. A. & Peterson, A. T. Modeling ecological niches and predicting geographic distributions: A test of six presence-only methods. *Rev. Mex. Biodivers.* 79, 205–216 (2008).
31. Elith, J. & Leathwick, J. R. Species distribution models: Ecological explanation and prediction across space and time. *Annu. Rev. Ecol. Evol. Syst.* 40, 677–697 (2009).<https://doi.org/10.1146/annurev.ecolsys.110308.120159>.
32. Sallam, M. F., Xue, R. D., Pereira, R. M. & Koehler, P. G. Ecological niche modeling of mosquito vectors of West Nile virus in St. John’s County, Florida, USA. *Parasit. Vectors* 9, 371 (2016).<https://doi.org/10.1186/s13071-016-1656-5>.
33. Li, S. L. *et al.* Mapping environmental suitability of *Haemagogus* and *Sabethes* spp. mosquitoes to understand sylvatic transmission risk of yellow fever virus in Brazil. *PLoS Negl. Trop. Dis.* 16, e0010019 (2022).<https://doi.org/10.1371/journal.pntd.0010019>.
34. Acosta, A. L., Giannini, T. C., Imperatriz-Fonseca, V. L. & Saraiva, A. M. Worldwide alien invasion: A methodological approach to forecast the potential spread of a highly invasive pollinator. *PLoS ONE* 11, e0148295 (2016).
35. Hao, T., Elith, J., Guillera-Arroita, G. & Lahoz-Monfort, J. J. A review of evidence about use and performance of species distribution modelling ensembles like BIOMOD. *Divers. Distrib.* 25, 839–852 (2019).<https://doi.org/10.1111/ddi.12892>.
36. Li, X. & Wang, Y. Applying various algorithms for species distribution modelling. *Integr. Zool.* 8, 124–135 (2013).<https://doi.org/10.1111/1749-4877.12000>.
37. Ryan, S. J. *et al.* Mapping current and future thermal limits to suitability for malaria transmission by the invasive mosquito *Anopheles stephensi*. *Malar. J.* 22, 45 (2023).<https://doi.org/10.1186/s12936-023-04531-4>.
38. McBride, L. A. *et al.* Comparison of CMIP6 historical climate simulations and future projected warming to an empirical model of global climate. *Earth Syst. Dyn.* 12, 545–579 (2021).
39. O’Neill, B. C. *et al.* The Scenario Model Intercomparison Project (ScenarioMIP) for CMIP6. *Geosci. Model Dev.* 9, 3461–3482 (2016).<https://doi.org/10.5194/gmd-9-3461-2016>.
40. Muschelli, J. rscopus: R Interface to Elsevier Scopus APIs. R package version 0.6.5 (2023). Available at:<https://cran.r-project.org/web/packages/rscopus/index.html>.
41. Boucher, O. *et al.* IPSL IPSL-CM6A-LR-INCA model output prepared for CMIP6 AerChemMIP. *Earth Syst. Grid Fed.* (2020).
42. Yukimoto, S. *et al.* MRI MRI-ESM2.0 model output prepared for CMIP6 AerChemMIP. *Earth Syst. Grid Fed.*<https://doi.org/10.22033/ESGF/CMIP6.633> (2019).
43. Takemura, T. MIROC MIROC6 model output prepared for CMIP6 AerChemMIP. Version 20230720. *Earth Syst. Grid Fed.* (2019).
44. Hay, S. I. & Snow, R. W. The Malaria Atlas Project: Developing Global Maps of Malaria Risk. *PLoS Med.* 3, e473 (2006).
45. Sinka, M. E. *et al.* A new malaria vector in Africa: Predicting the expansion range of *Anopheles stephensi* and identifying the urban populations at risk. *Proc. Natl. Acad. Sci. U.S.A.* 117, 24900–24908 (2020).
46. Tadesse, F. G. *et al.* *Anopheles stephensi* mosquitoes as vectors of *Plasmodium vivax* and *falciparum*, Horn of Africa, 2019. *Emerg. Infect. Dis.* 27, 603–607 (2021).
47. Balkew, M. *et al.* Geographical distribution of *Anopheles stephensi* in eastern Ethiopia. *Parasites Vectors* 13, 1–8 (2020).
48. Chefaoui, R. M. & Lobo, J. M. Assessing the effects of pseudo-absences on predictive distribution model performance. *Ecol. Model.* 210, 478–486 (2008).<https://doi.org/10.1016/j.ecolmodel.2007.08.010>.
49. Barbet-Massin, M., Jiguet, F., Albert, C. H. & Thuiller, W. Selecting pseudo-absences for species distribution models: how, where and how many? *Methods Ecol. Evol.* 3, 327–338 (2012).<https://doi.org/10.1111/j.2041-210X.2011.00172.x>.
50. Midega, J. T. *et al.* Estimating dispersal and survival of *Anopheles gambiae* and *Anopheles funestus* along the Kenyan Coast by using mark–release–recapture methods. *J. Med. Entomol.* 44, 923–929 (2007). [https://doi.org/10.1603/0022-2585(2007)44[923:edasoa]2.0.co;2](https://doi.org/10.1603/0022-2585(2007)44%5B923:edasoa%5D2.0.co;2).
51. Kaufmann, C. & Briegel, H. Flight performance of the malaria vectors *Anopheles gambiae* and *Anopheles atroparvus*. *J. Vector Ecol.* 29, 140–153 (2004).
52. Cho, S.-H. *et al.* A mark-release-recapture experiment with *Anopheles sinensis* in the northern part of Gyeonggi-do, Korea. *Korean J. Parasitol.* 40, 139 (2002).<https://doi.org/10.3347/kjp.2002.40.3.139>.
53. Santika, T. Assessing the effect of prevalence on the predictive performance of species distribution models using simulated data. *Glob. Ecol. Biogeogr.* 20, 181–192 (2010).<https://doi.org/10.1111/j.1466-8238.2010.00581.x>.
54. Meynard, C. N. & Kaplan, D. M. The effect of a gradual response to the environment on species distribution modeling performance. *Ecography* 35, 499–509 (2011).<https://doi.org/10.1111/j.1600-0587.2011.07157.x>.
55. Aguirre-Gutiérrez, J., Carvalheiro, L. G., WallisDeVries, M. F., Reemer, M. & Biesmeijer, J. C. Fit-for-purpose: Species distribution model performance depends on evaluation criteria – Dutch hoverflies as a case study. *PLoS ONE* 8, e63708 (2013).
56. Banfield, R. E., Hall, L. O., Bowyer, K. W. & Kegelmeyer, W. P. Ensemble diversity measures and their application to thinning. *Inf. Fusion* 6, 49–62 (2005).<https://doi.org/10.1016/j.inffus.2004.04.005>.
57. Minowa, Y. Verification for generalizability and accuracy of a thinning-trees selection model with the ensemble learning algorithm and the cross-validation method. *J. For. Res.* 13, 275–285 (2008).<https://doi.org/10.1007/s10310-008-0084-6>.
58. Aiello-Lammens, M. E., Boria, R. A., Radosavljevic, A., Vilela, B. & Anderson, R. P. spThin: an R package for spatial thinning of species occurrence records for use in ecological niche models. *Ecography* 38, 541–545 (2015).
59. Allouche, O., Tsoar, A. & Kadmon, R. Assessing the accuracy of species distribution models: prevalence, kappa and the true skill statistic (TSS). *J. Appl. Ecol.* 43, 1223–1232 (2006).<https://doi.org/10.1111/j.1365-2664.2006.01214.x>.
60. Naimi, B., Hamm, N. A. S., Groen, T. A., Skidmore, A. K. & Toxopeus, A. G. Where is positional uncertainty a problem for species distribution modelling? *Ecography* 37, 191–203 (2014).
61. Grenouillet, G., Buisson, L., Casajus, N. & Lek, S. Ensemble modelling of species distribution: The effects of geographical and environmental ranges. *Ecography* 34, 9–17 (2011).<https://doi.org/10.1111/j.1600-0587.2010.06152.x>.
62. World Health Organization. World malaria report 2023. World Health Organization, Geneva, Switzerland (2023).
63. Liu, C., White, M. & Newell, G. Selecting thresholds for the prediction of species occurrence with presence-only data. *J. Biogeogr.* 40, 778–789 (2013).<https://doi.org/10.1111/jbi.12058>.
64. Rousseau, J. S. & Betts, M. G. Factors influencing transferability in species distribution models. *Ecography* 2022, e06060 (2022).<https://doi.org/10.1111/ecog.06060>.
65. Barry, S. & Elith, J. Error and uncertainty in habitat models. *J. Appl. Ecol.* 43, 413–423 (2006).<https://doi.org/10.1111/j.1365-2664.2006.01136.x>.
66. Araújo, M. & New, M. Ensemble forecasting of species distributions. *Trends Ecol. Evol.* 22, 42–47 (2007).
67. Jiménez-Valverde, A. & Lobo, J. M. Threshold criteria for conversion of probability of species presence to either–or presence–absence. *Acta Oecol.* 31, 361–369 (2007).
68. Hijmans, R. J. *et al.* raster: Geographic data analysis and modeling. R package version 3.5-11 (2022). Available at:<https://cran.r-project.org/package=raster>.
69. Wickham, H. *et al.* dplyr: A Grammar of Data Manipulation. R package version 1.1.0 (2022). Available at:<https://cran.r-project.org/package=dplyr>.
70. Bischl, B. *et al.* pbapply: Adding Progress Bar to ‘apply’ Functions. R package version 1.6-0 (2022). Available at:<https://cran.r-project.org/package=pbapply>.
71. Wickham, H. ggplot2: Elegant Graphics for Data Analysis. Springer-Verlag New York (2016). R package version 3.4.4.
72. Pebesma, E. J. & Bivand, R. S. Classes and methods for spatial data in R. *R News* 5, 9–13 (2005). R package version 1.6-0. Available at:<https://cran.r-project.org/package=sp>.
