# Supplementary figures and images for: Future global distribution and climatic suitability of Anopheles stephensi
Source: Sci Rep. 2025 Jul 1;15:22268. doi: 10.1038/s41598-025-07653-8 (PMC12215484; doi:10.1038/s41598-025-07653-8)

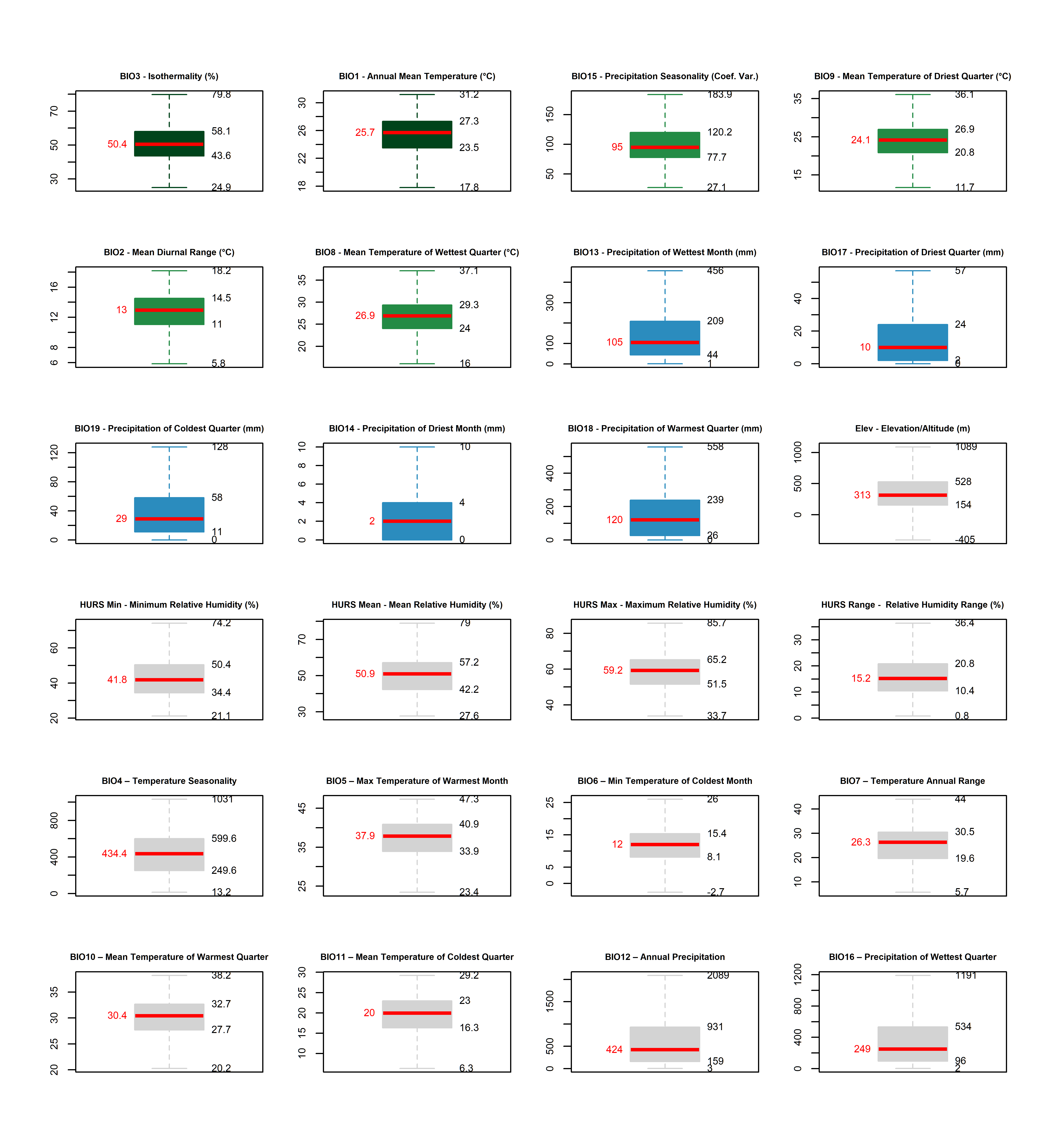

Supplement: Supplementary file 2 — Supplementary material 2 (PNG 327.1 kb) [file 41598_2025_7653_MOESM2_ESM.png]

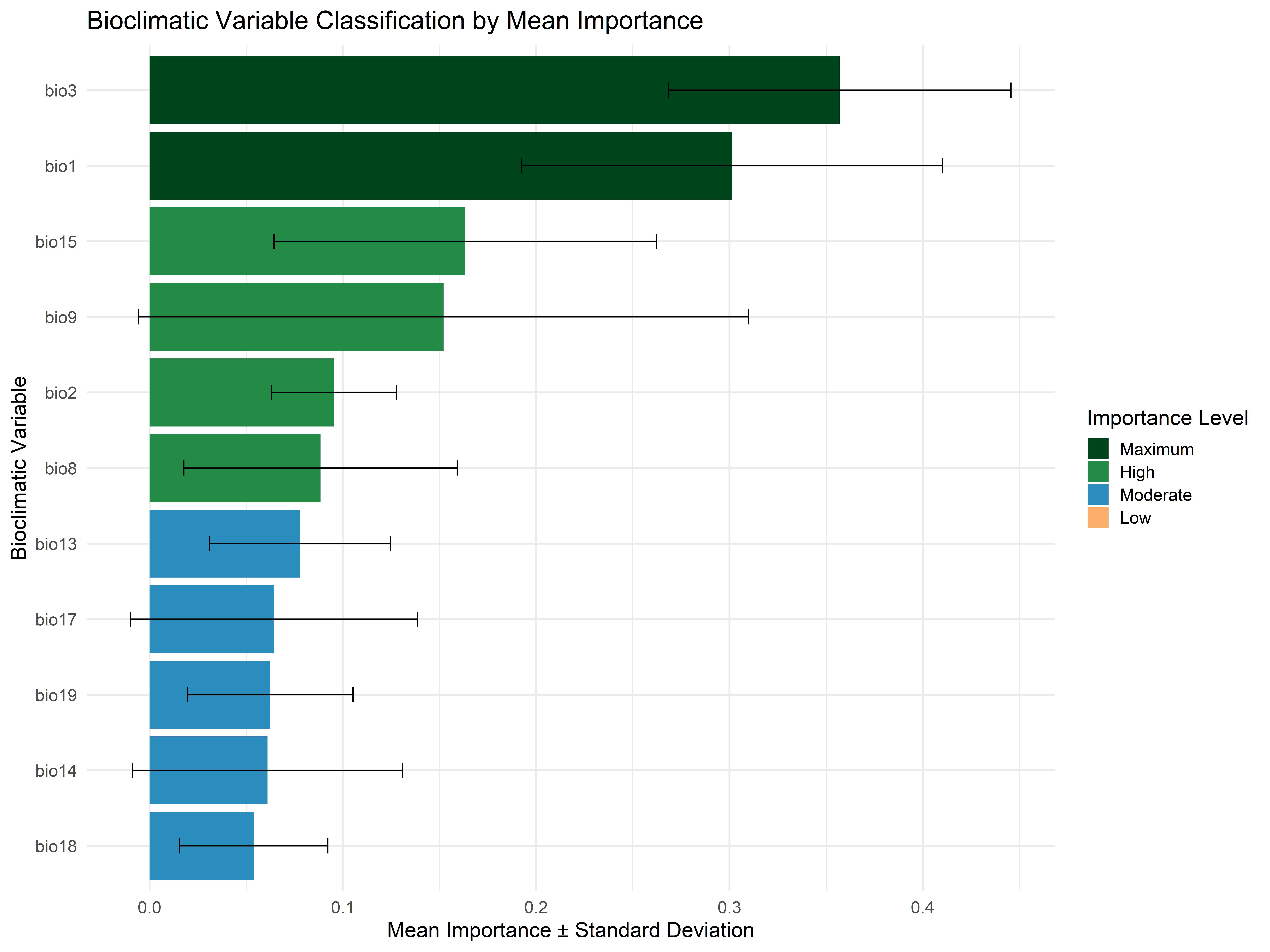

Supplement: Supplementary file 3 — Supplementary material 3 (PNG 266.3 kb) [file 41598_2025_7653_MOESM3_ESM.png]

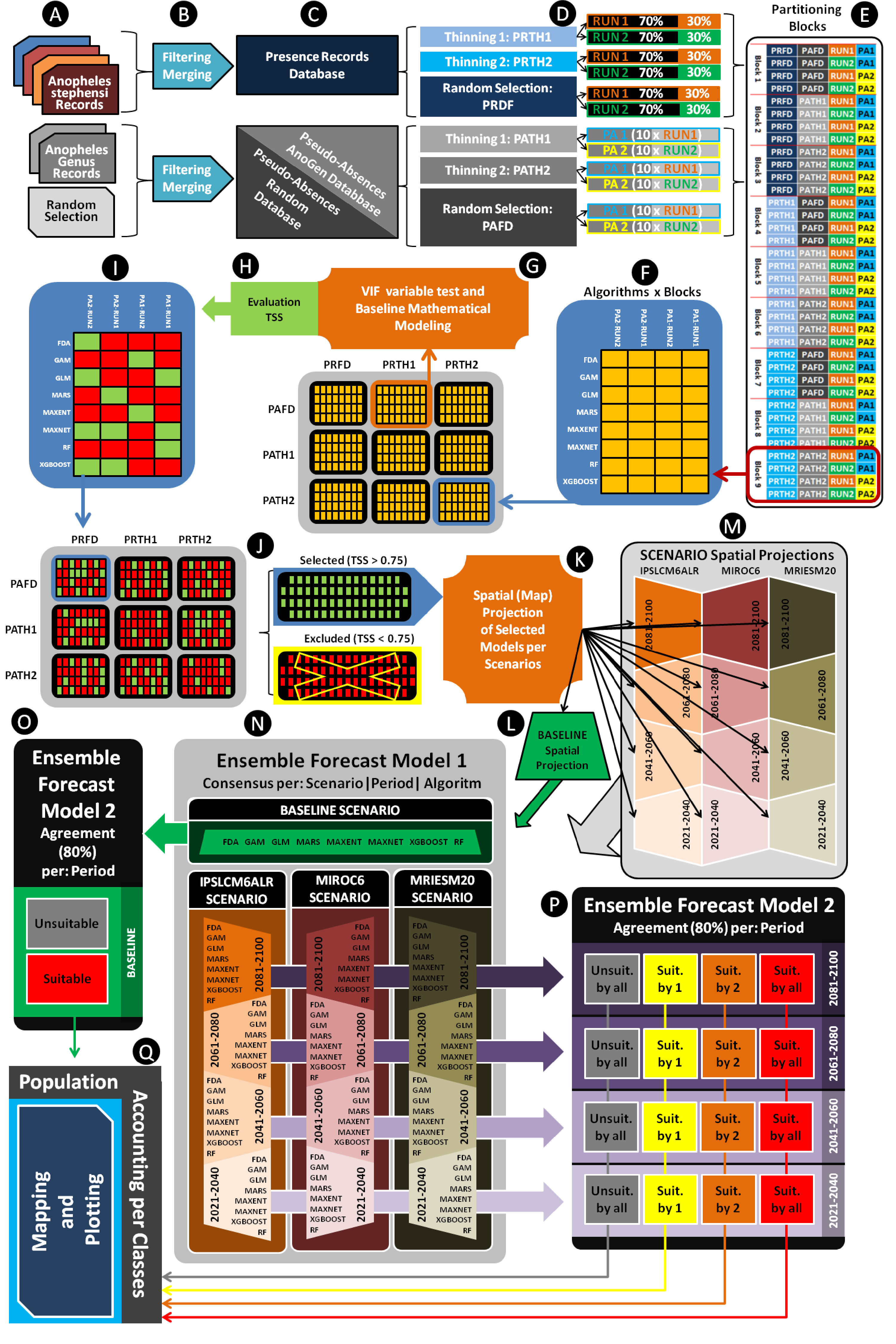

Supplement: Supplementary file 4 — Supplementary material 4 (PNG 1001.9 kb) [file 41598_2025_7653_MOESM4_ESM.png]

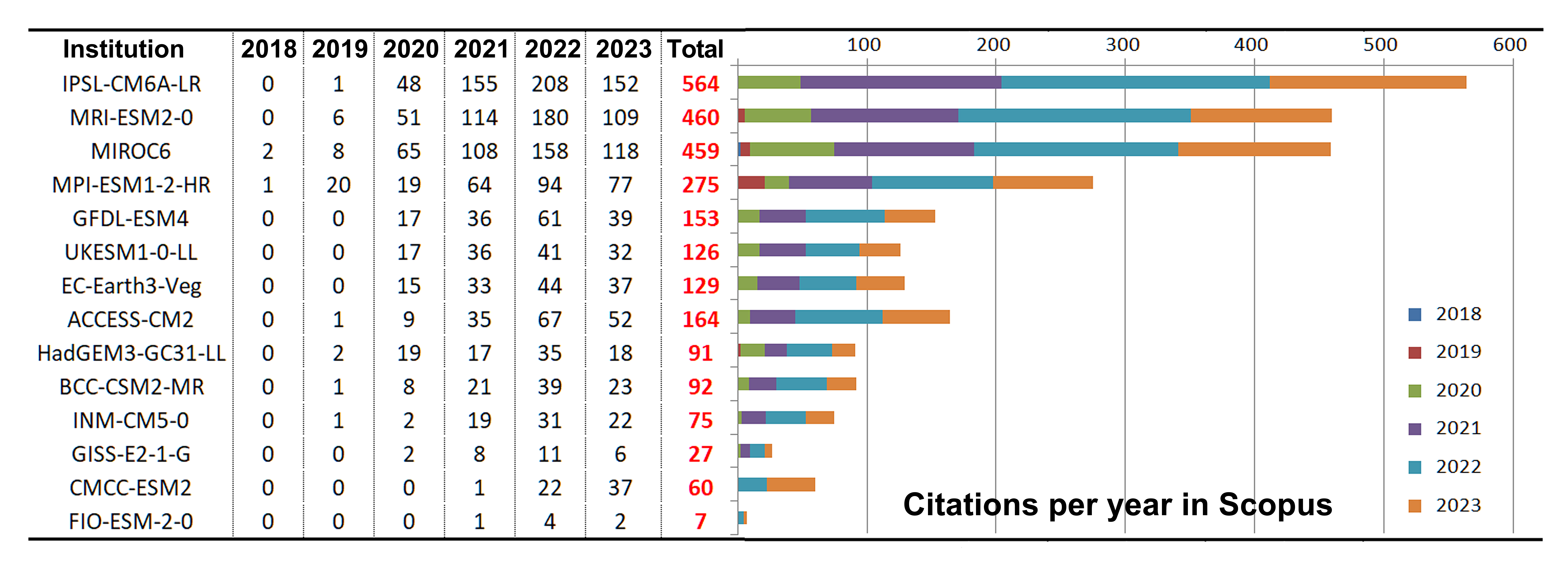

Supplement: Supplementary file 6 — Supplementary material 6 (PNG 3338.9 kb) [file 41598_2025_7653_MOESM6_ESM.png]

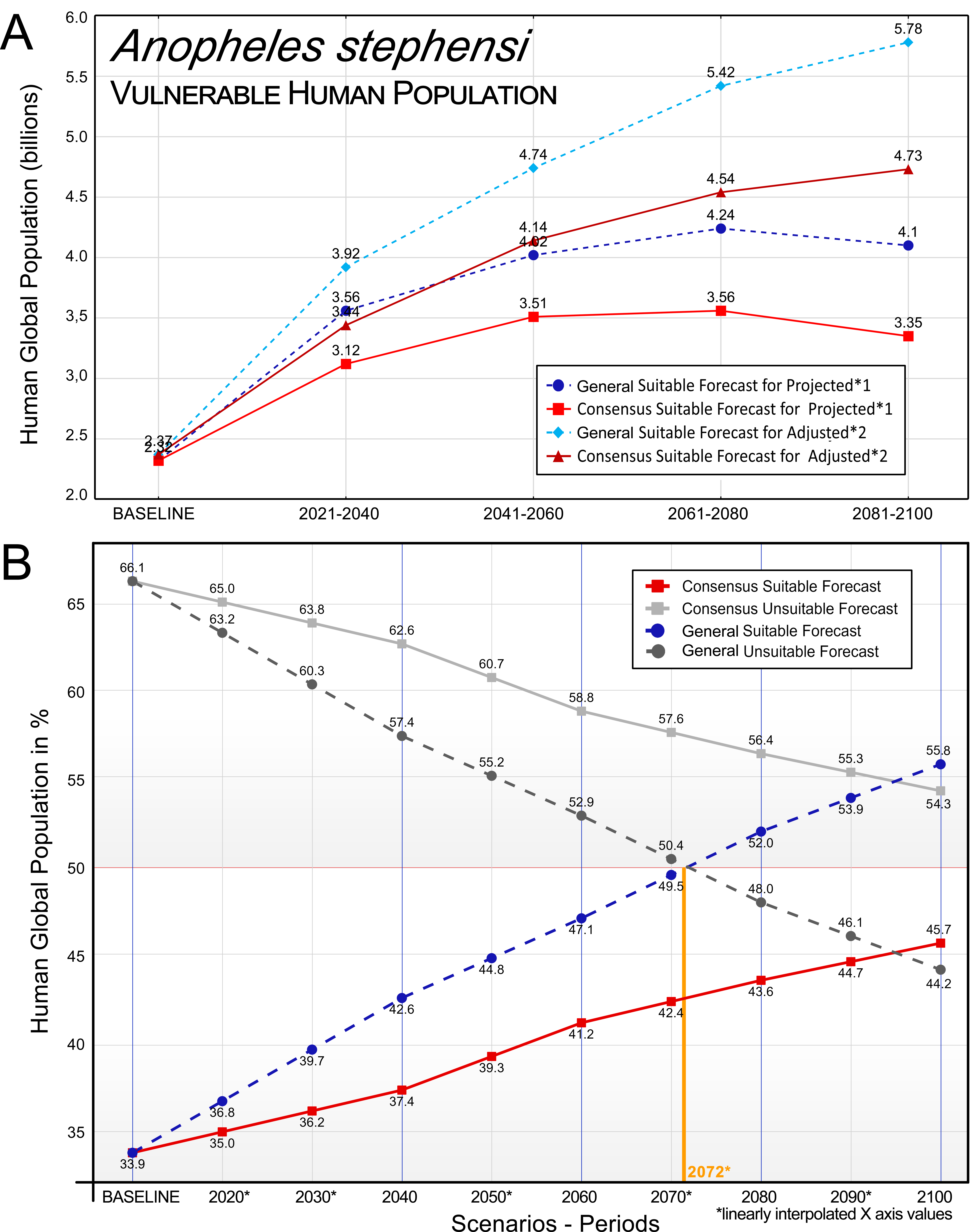

Supplement: Supplementary file 7 — Supplementary material 7 (PNG 2243.3 kb) [file 41598_2025_7653_MOESM7_ESM.png]

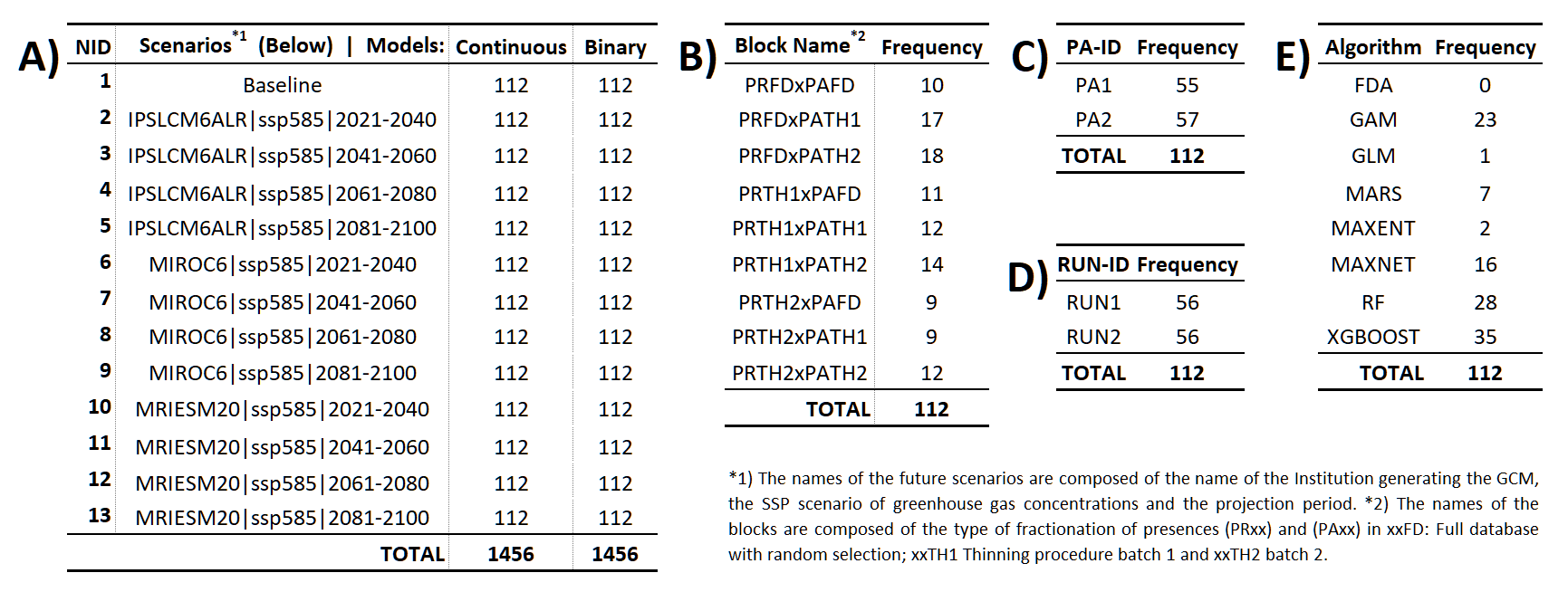

Supplement: Supplementary file 10 — Supplementary material 10 (PNG 86.3 kb) [file 41598_2025_7653_MOESM10_ESM.png]
